# Strains of *Bradyrhizobium cosmicum* sp. nov., isolated from contrasting habitats in Japan and Canada possess photosynthesis gene clusters with the hallmark of genomic islands

**DOI:** 10.1099/ijsem.0.004380

**Published:** 2020-08-17

**Authors:** Sawa Wasai-Hara, Kiwamu Minamisawa, Sylvie Cloutier, Eden S. P. Bromfield

**Affiliations:** ^1^​ Graduate School of Life Sciences, Tohoku University, Katahira, Aoba-ku, Sendai 980–8577, Japan; ^2^​ Ottawa Research and Development Centre, Agriculture and Agri-Food Canada, 960 Carling Avenue, Ottawa, Ontario K1A OC6, Canada

**Keywords:** *Bradyrhizobium cosmicum*, complete genome sequence, genomic island, photosynthesis gene cluster

## Abstract

The taxonomic status of two previously characterized *Bradyrhizobium* strains (58S1^T^ and S23321) isolated from contrasting habitats in Canada and Japan was verified by genomic and phenotypic analyses. Phylogenetic analyses of five and 27 concatenated protein-encoding core gene sequences placed both strains in a highly supported lineage distinct from named species in the genus *Bradyrhizobium* with *Bradyrhizobium betae* as the closest relative. Average nucleotide identity values of genome sequences between the test and reference strains were between 84.5 and 94.2 %, which is below the threshold value for bacterial species circumscription. The complete genomes of strains 58S1^T^ and S23321 consist of single chromosomes of 7.30 and 7.23 Mbp, respectively, and do not have symbiosis islands. The genomes of both strains have a G+C content of 64.3 mol%. Present in the genome of these strains is a photosynthesis gene cluster (PGC) containing key photosynthesis genes. A tRNA gene and its partial tandem duplication were found at the boundaries of the PGC region in both strains, which is likely the hallmark of genomic island insertion. Key nitrogen-fixation genes were detected in the genomes of both strains, but nodulation and type III secretion system genes were not found. Sequence analysis of the nitrogen fixation gene, *nifH*, placed 58S1^T^ and S23321 in a novel lineage distinct from described *Bradyrhizobium* species. Data for phenotypic tests, including growth characteristics and carbon source utilization, supported the sequence-based analyses. Based on the data presented here, a novel species with the name *Bradyrhizobium cosmicum* sp. nov. is proposed with 58S1^T^ (=LMG 31545^T^=HAMBI 3725^T^) as the type strain.

The genus *Bradyrhizobium* is a large and diverse group of bacterial species and includes members that possess accessory genes for nitrogen fixation, photosynthesis and/or symbiotic interaction with legume plants [1].

In a previous study [2], bacteria were isolated from root nodules of soybean plants that had been inoculated with root-zone soils from legumes native to Canada. Bacterial isolates were characterized by multiple locus sequence analysis (MLSA) of five protein-encoding core genes and several novel lineages in the genus *Bradyrhizobium* were identified. One of these novel lineages, represented by strain 58S1^T^, is a close relative of *Bradyrhizobium betae* PL7HG1^T^ [3] that was recently reported to harbour key photosystem genes [4].

During the course of the present work we showed that strain 58S1^T^ also possesses photosystem genes and, based on results of taxonomic analyses, is highly similar to *Bradyrhizobium* sp. S23321 [5], which was isolated from paddy field soil in Japan and has been subjected to detailed genomic analysis.

Here we used complete genome, phylogenetic and phenotypic analyses to further characterize strains 58S1^T^ and S23321 and based on the results a novel species for which the name *Bradyrhizobium cosmicum* sp. nov. is proposed.

## Habitat and isolation

Novel strain 58S1^T^ was isolated from a root nodule of a soybean plant that had been inoculated with a suspension of root-zone soil of *Amphicarpaea bracteata* (hog peanut) plants growing in deciduous woodland in Gatineau, Quebec, Canada [2]. Strain 58S1^T^ was deposited in the BCCM/LMG Bacteria Collection, University of Ghent, Belgium (LMG collection no. 31545) and in the HAMBI Microbial Culture Collection, University of Helsinki, Finland (HAMBI collection no. 3725). Novel strain S23321 was isolated from paddy field soil at the experimental farm of Tohoku University, Japan [5] and was deposited in the Japan Collection of Microorganisms (JCM collection no. 18004).

## Phylogenetic characterization of partial gene sequences

For phylogenetic analyses, sequences of 16S rRNA, *atpD*, *glnII*, *gyrB*, *recA* and *rpoB* core genes were used. Nucleotide sequence accession numbers are given in Table S1 (available in the online version of this article). Sequence alignments of protein-encoding core genes (*atpD*, *glnII*, *gyrB*, *recA* and *rpoB*) were carried out as previously described [6]. Alignment of 16S rRNA gene sequences was done using the fast, secondary-structure aware Infernal aligner version 1.1 using the online Ribosomal Database Project version 11.5 [7]. Best-fit substitution models were selected using ModelTest-NG [8] implemented in the cipres Science Gateway version 3.3 [9]. Maximum-likelihood (ML) phylogenetic analyses [10] were performed using 1000 non-parametric bootstrap replications to assess support [6]. Bayesian phylogenetic analyses were carried out using MrBayes version 3.2.1 with default priors [11] as detailed previously [12]. In all instances, tree topologies from Bayesian and ML analyses were similar and therefore only the Bayesian trees are shown.

In order to include all described species of the genus *Bradyrhizobium* in a phylogenetic analysis of 16S rRNA gene sequences, it was necessary to trim aligned sequence lengths to 1300 bp. The 16S rRNA gene tree (Fig. S1) shows that novel strains 58S1^T^ and S23321 possess identical 16S rRNA gene sequences and were placed in a superclade represented by *Bradyrhizobium japonicum* with the type strain of *B. betae* their as closest relative. Sequence similarities of the 16S rRNA gene of these *Bradyrhizobium* species versus 58S1^T^ and S23321 (Table S2), calculated using software implemented in EzBioCloud [13], are consistent with the phylogenetic data indicating that *B. betae* is the closest relative. It should be noted, however, that the 16S rRNA gene is highly conserved and its usefulness as a taxonomic marker for species delineation in the genus *Bradyrhizobium* is limited [14, 15].

**Fig. 1.**
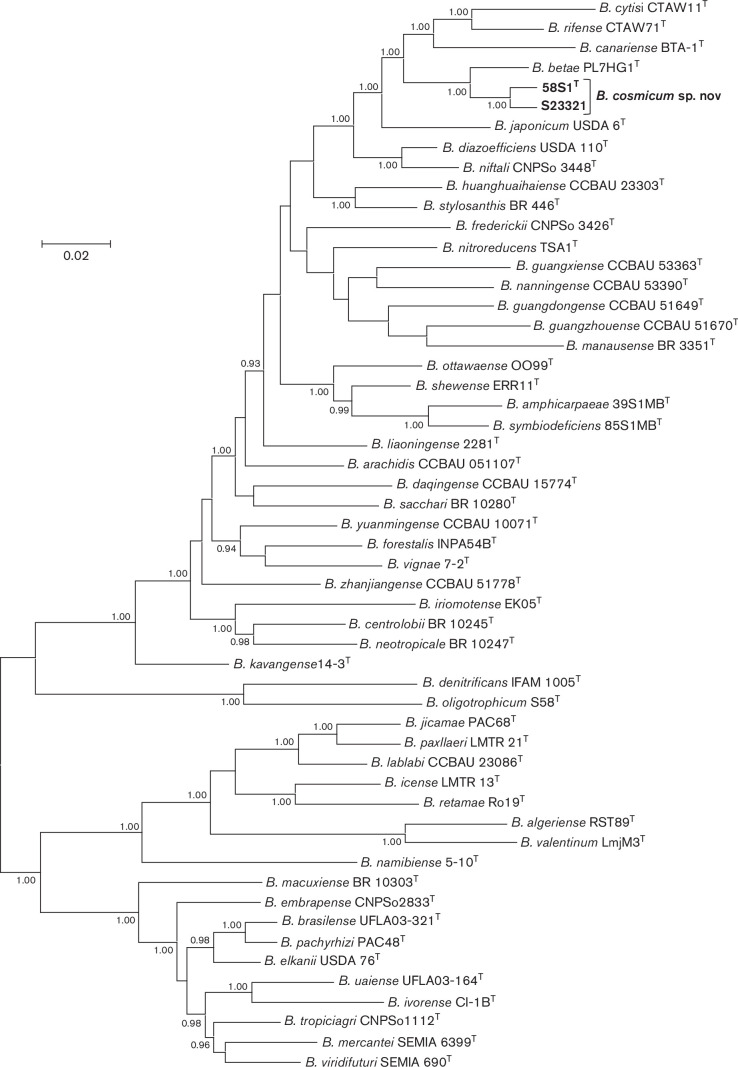
Bayesian phylogenetic tree (GTR+G+I substitution model) of *atpD–glnII–recA–gyrB–rpoB* concatenated housekeeping gene sequences for *Bradyrhizobium cosmicum* sp. nov. and reference taxa of the genus *Bradyrhizobium *. Alignment lengths: *atpD*, 429 bp; *glnII,* 519 bp; *recA,* 417 bp; *gyrB*, 600 bp; *rpoB,* 714 bp; total, 2679 bp. Posterior probabilities ≥0.90 are shown. Bar, expected substitutions per site.

MLSA of five or more core gene sequences represents a widely used and reliable method for phylogenetic analysis and delineation of species within the genus *Bradyrhizobium* [6, 15–17]. The Bayesian tree of five concatenated protein-encoding core gene sequences (Fig. 1) placed strains 58S1^T^ and S23321 in a highly supported lineage distinct from described *Bradyrhizobium* species with *B. betae* PL7HG1^T^ as the closest relative. Similar results were obtained for the trees of individual core gene sequences (Figs S2–S6).

As one or more core gene sequences of type strains of several *Bradyrhizobium* species are not available in public databases, we carried out a supplementary phylogenetic analysis using the only two gene sequences (*recA* and *glnII*) that are available for all described species. In order to include all type strains of these species in the analysis, it was necessary to trim the aligned sequence lengths to 411 and 519 bp for the *recA* and *glnII* genes, respectively. The topology of the Bayesian tree of concatenated *recA–glnII* gene sequences (Fig. S7) corroborates the placement of novel strains 58S1^T^ and S23321 in a lineage distinct from named species of the genus *Bradyrhizobium *.

Percentage sequence similarities for 58S1^T^ and S23321 versus type strains of reference taxa for the five concatenated core gene sequences, calculated by the method of Stothard [18] (Table S2), were at or below the threshold value of ~97% proposed for species differentiation in the genus *Bradyrhizobium* [19].

**Fig. 2.**
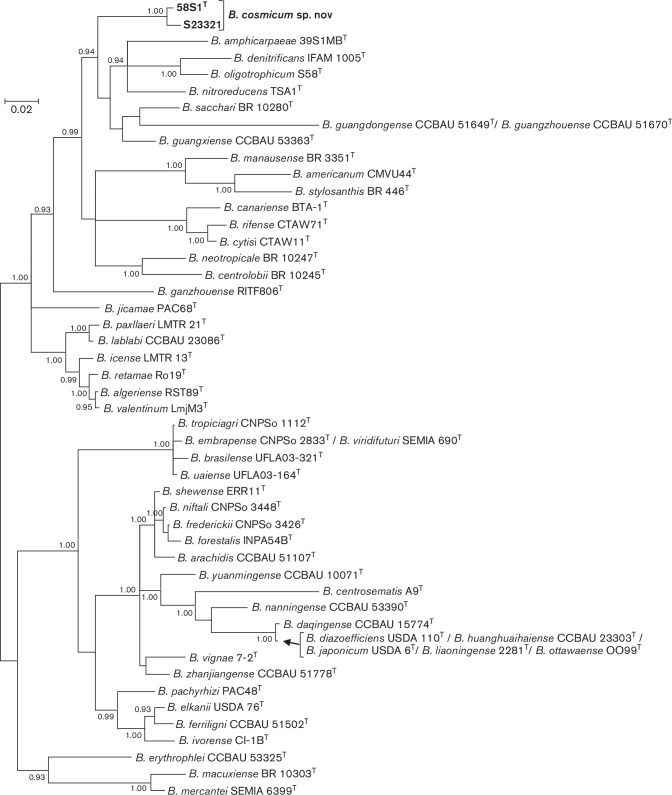
Bayesian phylogenetic tree (HKY+G substitution model) of *nifH* gene sequences (540 bp) for *Bradyrhizobium cosmicum* sp. nov. and reference taxa of the genus *Bradyrhizobium *. Posterior probabilities ≥0.90 are shown. Bar, expected substitutions per site.

A Bayesian tree of partial sequences of the *nifH* gene for 58S1^T^ and S23321 and type strains of *Bradyrhizobium* species is given in Fig. 2. The *nifH* gene tree shows that 58S1^T^ and S23321 are placed in a lineage that is distinct from described *Bradyrhizobium* species. Closest relatives include type strains of *Bradyrhizobium amphicarpaeae *, *Bradyrhizobium oligotrophicum* and *Bradyrhizobium denitrificans* that possess photosystem genes and *Bradyrhizobium guangxiense, Bradyrhizobium nitroreducens* and *Bradyrhizobium sacchari* that do not possess photosystem genes. Sequence similarity values for the *nifH* gene of 58S1^T^ and S23321 in pairwise comparisons with *Bradyrhizobium* reference taxa were between 83.5 and 93.0 % (Table S2).

## Genomic characterization

The complete genome of strain 58S1^T^ was sequenced at the Genome Quebec Innovation Centre, Montreal, Canada, using the Pacific Biosciences (PacBio) RS II single-molecule real-time (SMRT) platform [20] as described previously [21]. Estimated genome coverage for strain 58S1^T^ was 136-fold with 104 918 polymerase reads and an average read length of 13 299 bp. The complete genome sequence of S23321 was determined in a previous study [5] by a whole genome shotgun approach using hybrid assembly of Sanger end sequences consisting of 3 kb and 10 kb clone libraries (53 760 reads, 4.5-fold genome coverage) and 454 pyrosequencing data with appropriate gap filling procedures.

The complete genomes of strains 58S1^T^ and S23321 consist of single circular chromosomes of size 7 304 136 bp and 7 231 841 bp, respectively. These genomes are similar in size to close relative, *B. betae* PL7HG1^T^, with a complete genome of size 7 419 402 bp (Table 1). However, unlike *B. betae* PL7HG1^T^, novel strains 58S1^T^ and S23321 do not possess plasmids [4].

**Table 1.** Characteristics of genome sequences of *Bradyrhizobium cosmicum* sp. nov., strains 58S1^T^ (accession no. CP041656) and S23321 (accession no. AP012279) and close relatives, *Bradyrhizobium betae* PL7HG1^T^ (accession no. CP044543), *Bradyrhizobium diazoefficiens* USDA 110^T^ (accession no. CP011360) and *Bradyrhizobium japonicum* USDA 6^T^ (accession no. AP012206) Unless otherwise stated, data are from the NCBI assembly databases. na, Data not available.

Characteristic	Strain				
	58 S1^T^	S23321	PL7HG1^T^	USDA110^T^	USDA6^T^
Genome assembly quality	Complete	Complete	Complete	Complete	Complete
Genome size (bp)	7 304 136	7 231 841	7 419 402	9 105 828	9 207 384
CDS (total)	6930	6983*	7113	8489	9447*
CDS (coding)	6757	6898†	6780	8220	8829‡
rRNAs	3	3	3	3	6‡
tRNAs	48	45†	47	52	51‡
Pseudo genes (total)	173	122†	333	213	na
Repeat regions	58*	na	58*	148*	128*
DNA G+C content (mol%)	64.3*	64.3†	64.8§	64.1*	63.7‡
Photosynthesis gene cluster	Yes	Yes	Yes	No	No
Plasmids	0	0	1	0	0
Symbiosis island	No	No	No	Yes	Yes

*Data from the patric Bioinformatics Database [24].

†Data from Okubo *et al*. 2012 [5].

‡Data from Kaneko *et al*. 2011 [49].

§Data from Cloutier and Bromfield 2019 [4].

The DNA G+C content of strains 58S1^T^ and S23321 is 64.3 mol%, which is within the range for members of the genus *Bradyrhizobium *. Totals of 6930 coding sequences, 48 tRNAs and a single rRNA operon were found for strain 58S1^T^ using the NCBI Prokaryotic Genome Annotation Pipeline version 4 [22, 23]. For strain S23321, 6983 coding sequences, 45 tRNAs and a single rRNA operon were detected using the patric version 3.5.26 platform [24].

The most abundant genes for strains 58S1^T^ and S23321, respectively, are those involved in metabolism (1045 and 1007 genes), energy (310 and 301 genes), protein processing (235 and 229 genes), membrane transport (231 and 246 genes) and cellular processes (175 and 160 genes). Genes involved in motility and chemotaxis, stress response (heat/cold and osmotic shock), resistance to antibiotics and toxic compounds were also detected in both 58S1^T^ and S23321.

Average nucleotide identity (ANI) as an overall genome relatedness index is recommended to replace DNA–DNA hybridization methods for bacterial species delineation [14, 15, 25, 26]. We estimated ANI values for the complete genome sequence of 58S1^T^ and S23321 in pairwise comparisons with genome sequences of type strains of described *Bradyrhizobium* species available in public databases using the MUMmer (ANIm) algorithm implemented in the J-species web server version 3.0.20 [27]. Table 2 shows that, compared to 58S1^T^ and S23321, ANI values varied between 84.4 % (* B. retamae* Ro19^T^) and 94.2 % (* B. betae* PL7HG1^T^), which is below the accepted threshold value of 95–96  % for bacterial species circumscription [14, 25, 28]. In contrast, the ANI value of 97.9% for the comparison of novel strains 58S1^T^ versus S23321 is consistent with these strains belonging to the same species. These data are also in accord with the phylogenetic results (Fig. 1) indicating that *B. betae* PL7HG1^T^ is a close relative of novel strains 58S1^T^ and S23321.

**Table 2.** Average nucleotide identity (ANI) values for pairwise comparisons of genome sequences of *Bradyrhizobium cosmicum* sp. nov 58S1^T^ and S23321 versus *Bradyrhizobium* species in public databases

Reference strain (accession no.)	ANI (%)		Reference strain (accession no.)	ANI (%)	
	58 S1^T^	S23321		58 S1^T^	S23321
*** Bradyrhizobium cosmicum* 58 S1^T^** (**CP041656**)	–	**97.9**	* Bradyrhizobium guangdongense* CCBAU 51649^T^ (CP030051)	87.7	87.8
***Bradyrhizobiumcosmicum* S23321** (**AP012279**)	**97.9**	–	* Bradyrhizobium manausense* BR 3351^T^ (LJYG00000000)	87.7	87.7
* Bradyrhizobium betae* PL7HG1^T^ (CP044543)	94.1	94.2	*Bradyrhizobium centrolobii* BR 10245^T^ (LUUB00000000)	87.7	87.7
* Bradyrhizobium diazoefficiens* USDA 110^T^ (CP011360)	89.4	89.4	* Bradyrhizobium neotropicale* BR 10247^T^ (LSEF00000000)	87.6	87.6
* Bradyrhizobium japonicum* USDA 6^T^ (AP012206)	89.4	89.4	* Bradyrhizobium ivorense* CI-1B^T^ (CDFC00000000)	85.3	85.3
* Bradyrhizobium niftali* CNPSo 3448^T^ (SPQT00000000)	89.4	89.4	*Bradyrhizobium brasilense* UFLA03-321^T^ (MPVQ00000000)	85.3	85.3
* Bradyrhizobium. stylosanthis* BR 446^T^ (LVEM00000000)	88.8	88.9	* Bradyrhizobium elkanii* USDA76^T^ (ARAG01000000)	85.3	85.3
* Bradyrhizobium. arachidis* CCBAU 051107^T^ (FPBQ00000000)	88.8	88.8	* Bradyrhizobium mercantei* SEMIA 6399^T^ (MKFI00000000)	85.3	85.3
* Bradyrhizobium symbiodeficiens* 85S1MB^T^ (CP029427)	88.8	88.8	*Bradyrhizobium uaiense* UFLA03-164^T^ (VKHP00000000)	85.2	85.2
* Bradyrhizobium ottawaense* OO99^T^ (CP029425)	88.8	88.8	* Bradyrhizobium tropiciagri* CNPSo 1112^T^ (LFLZ00000000)	85.2	85.3
* Bradyrhizobium shewense* ERR11^T^ (FMAI00000000)	88.7	88.8	* Bradyrhizobium embrapense* CNPSo 2833^T^ (LFIP00000000)	85.2	85.2
* Bradyrhizobium amphicarpaeae* 39S1MB^T^ (CP029426)	88.6	88.6	* Bradyrhizobium pachyrhizi* PAC48^T^ (LFIQ00000000)	85.2	85.3
* Bradyrhizobium nitroreducens* TSA1^T^ (LFJC00000000)	88.6	88.6	* Bradyrhizobium viridifuturi* SEMIA 690^T^ (LGTB00000000)	85.2	85.2
* Bradyrhizobium frederickii* CNPSo 3426^T^ (SPQS00000000)	88.6	88.6	*Bradyrhizobium macuxiense* BR 10303^T^ (LNCU00000000)	85.1	85.1
* Bradyrhizobium zhanjiangense* CCBAU 51778^T^ (CP022221)	88.6	88.6	* Bradyrhizobium oligotrophicum* S58^T^ (AP012603)	84.8	84.9
*Bradyrhizobium forestalis* INPA54B^T^ (PGVG00000000)	88.6	88.6	* Bradyrhizobium lablabi* CCBAU 23086^T^ (LLYB00000000)	84.7	84.7
*Bradyrhizobium sacchari* BR 10280^T^ (LWIG00000000)	88.5	88.5	* Bradyrhizobium jicamae* PAC68^T^ (LLXZ00000000)	84.7	84.6
* Bradyrhizobium nanningense* CCBAU 53390^T^ (LBJC00000000)	88.4	88.4	* Bradyrhizobium paxllaeri* LMTR 21^T^ (MAXB00000000)	84.7	84.7
* Bradyrhizobium vignae* 7-2^T^ (RDQF00000000)	88.3	88.3	* Bradyrhizobium algeriense* RST89^T^ (PYCM00000000)	84.6	84.5
* Bradyrhizobium guangxiense* CCBAU 53363^T^ (CP022219)	88.3	88.3	* Bradyrhizobium valentinum* LmjM3^T^ (LLXX00000000)	84.5	84.5
* Bradyrhizobium yuanmingense* CCBAU10071^T^ (FMAE00000000)	88.2	88.2	* Bradyrhizobium icense* LMTR 13^T^ (CP016428)	84.5	84.5
* Bradyrhizobium guangzhouense* CCBAU 51670^T^ (CP030053)	87.8	87.8	* Bradyrhizobium retamae* Ro19^T^ (LLYA00000000)	84.4	84.5

**Fig. 3.**
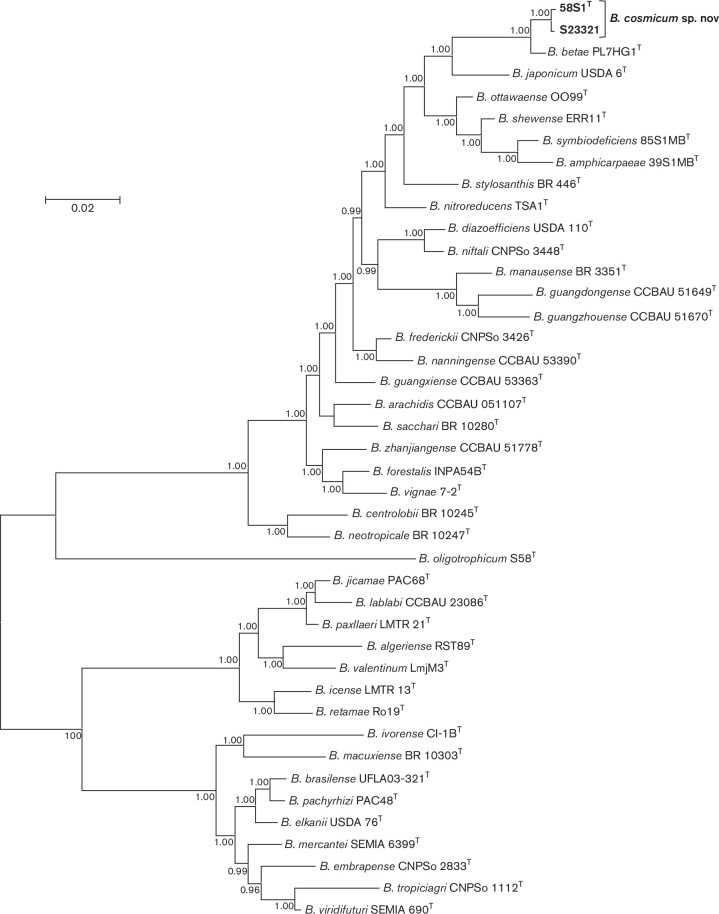
Bayesian phylogenetic tree (JTT +G+I substitution model) inferred from 27 concatenated protein encoding gene sequences for strains of *Bradyrhizobium cosmicum* sp. nov. and type strains of 41 *Bradyrhizobium* species. Alignment lengths (amino acids): *frr*, 191; *infC*, 196; *nusA*, 536; *pgk*, 394; *pyrG*, 545; *rplA*, 232; *rplB*, 196; *rplC*, 244; *rplE*, 201; *rplF*, 178; *rplK*, 142; *rplL*, 124; *rplM*, 170; *rplN*, 128; *rplP*, 141; *rplS*, 139; *rpmA*, 94; *rpoB*, 1030; *rpsB*, 342; *rpsC*, 237; *rpsE*, 193; *rpsI*, 158; *rpsK*, 130; *rpsM*, 131; *rpsS*, 96; *smpB*, 158; *tsf*, 308; total, 6634. Posterior probabilities ≥0.90 are shown. Bar, expected substitutions per site.

Phylogenomic relationships were investigated employing amino acid sequences of 27 conserved marker genes obtained from the genomes of 41 type strains of *Bradyrhizobium* species available in public databases using AmphoraNet [29], a web-based implementation of amphora2 [30]. Sequences were aligned with muscle [31] and then processed with TrimAl [32] to remove poorly aligned regions. Alignments were concatenated and the best-fit amino acid substitution model was selected using ModelTest-NG [8]. The placement of taxa in a Bayesian phylogenetic tree (Fig. 3) corroborates our finding that the closest relative of novel strains 58S1^T^ and S23321 is *B. betae* PL7HG1^T^. Fig. 3 also shows that the 41 *Bradyrhizobium* type strains were divided into four highly supported ‘superclades’ represented by type strains of *B. japonicum *, *B. oligotrophicum *, *B. elkanii* and *B. jicamae *, with novel strains 58S1^T^ and S23321 placed in the superclade represented by *B. japonicum *. These four superclades have also been identified in other phylogenetic studies of the genus *Bradyrhizobium* (e.g. [1, 33, 34]). It is noteworthy that the overall topology of the tree in Fig. 3 is consistent with the tree in Fig. 1, thereby validating the use of five concatenated core gene sequences for species delineation.

**Fig. 4.**
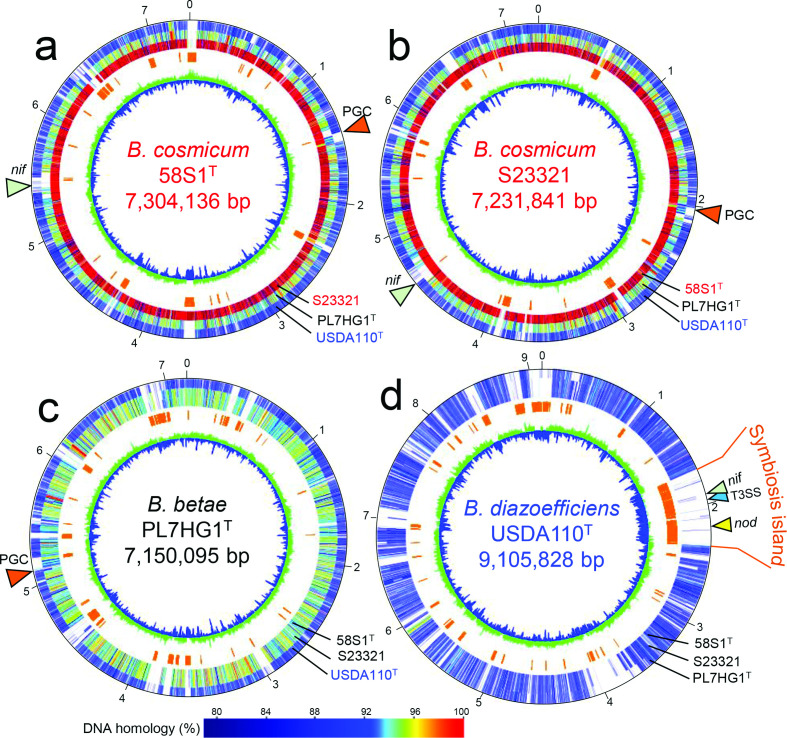
Circular representation of the chromosome of strains 58S1^T^ (a), S23321 (b), *Bradyrhizobium betae* PL7HG1^T^ (c) and *Bradyrhizobium diaozefficiens* USDA110^T^ (d). The innermost circle shows G+C content (blue and green indicate a value lower and higher than the average, respectively). The orange bars in the second inner circle denote genomic islands as predicted by IslandViewer 4 [41]. The inner third to fifth circles indicate the blastn homology (bl2seq, calculated by GenomeMatcher [35] with the respective strains of *Bradyrhizobium *, where the percent of blastn homology is indicated in the colour bar at the bottom. Numbers on outermost circles indicate genomic positions (Mb) on each chromosome. The position of the symbiosis island is shown on the circular presentation of *Bradyrhizobium diazoefficiens* USDA 110 [36, 49]. Orange, green, yellow and blue arrowheads show the positions of the photosynthesis gene cluster (PGC), *nif*, *nod* and type III secretion system (T3SS) genes, respectively.

Further genomic analysis of novel strains was done using GenomeMatcher software [35]. Circular representation of genomes (Fig. 4a–c) shows that strain 58S1^T^ has high similarity to S23321 throughout its genome whereas similarity of both novel strains to *B. betae* PL7HG1^T^ is relatively low. These observations are consistent with expectations for comparisons within and between *Bradyrhizobium* species. Comparison with the genome of soybean-nodulating bacterium *B. diazoefficiens* USDA 110^T^ [36], show that novel strains 58S1^T^ and S23321, like close relative *B. betae* PL7HG1^T^, do not contain a symbiosis island that carries nodulation genes (*nodDYABCSUIJ*) or type III secretion systems (T3SS) genes required for symbiotic interaction with leguminous plants (Fig. 4d, Table 1). However, key genes for nitrogen fixation, including *nifDKEN*, *nifH*, *nifA* and *fixABCX*, were detected in the genomes of 58S1^T^ and S23321 but not in the genome of *B. betae* PL7HG1^T^. It is noteworthy that the organization of the *nif–fix* gene cluster is highly conserved in novel strains 58S1^T^ and S23321 (Fig. S8).

**Fig. 5.**
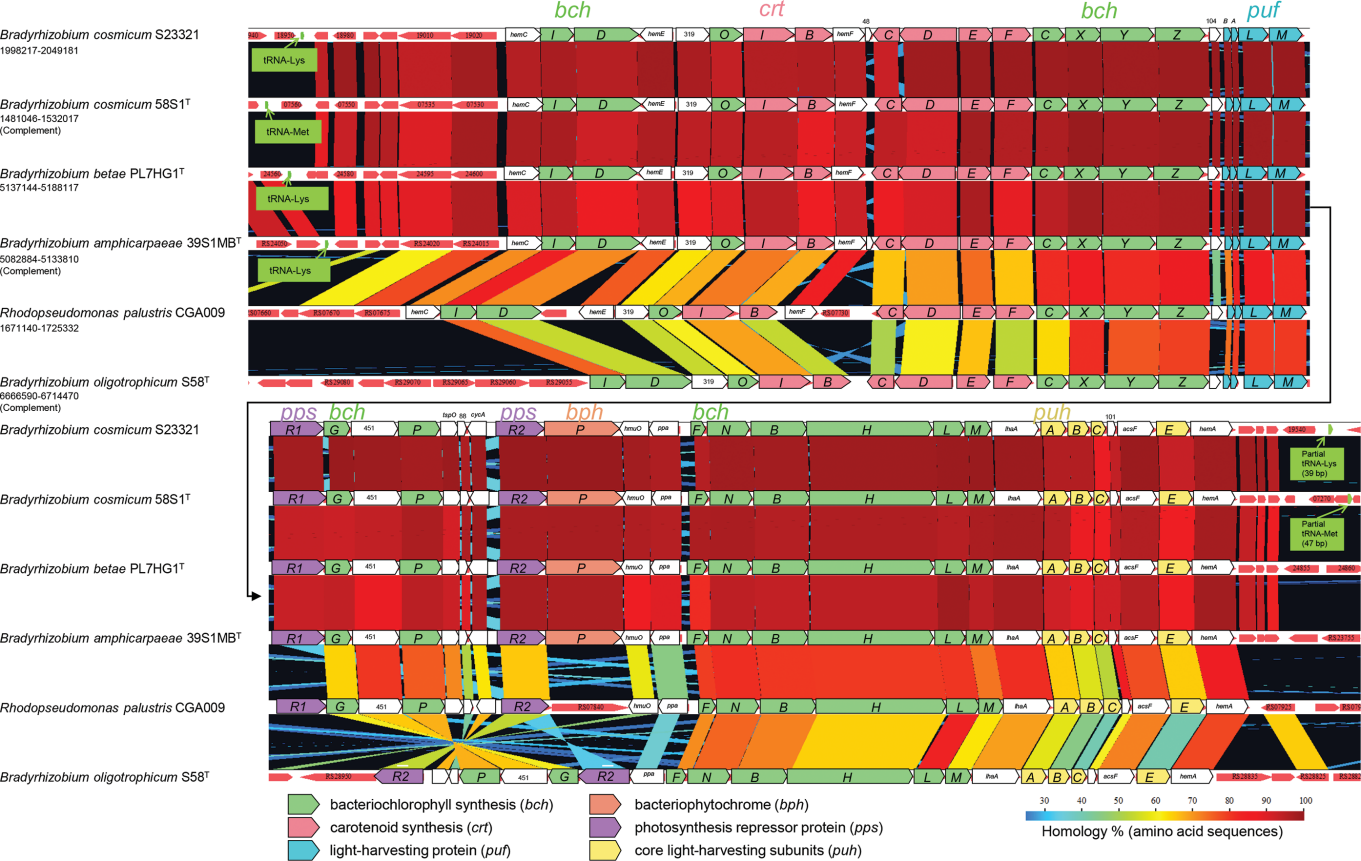
Comparative arrangement of the photosynthesis gene cluster (PGC) among *Bradyrhizobium cosmicum* 58S1^T^ and S23321, *Bradyrhizobium betae* PL7HG1^T^, *Bradyrhizobium amphicarpaeae* 39S1MB^T^, *Bradyrhizobium oligotrophicum* S58^T^ and *Rhodopseudomonas palustris* CGA009. Colour coding represents % homology based on amino acid sequences of PGC genes calculated by GenomeMatcher [35].

Further analyses show that the genomes of strains 58S1^T^ and S23321 [5] contain, respectively, a photosynthesis gene cluster (PGC) of about 51 kb (coordinates, 1 481046–1 532017 bp and 1998217–2 049181 bp). The PGC in both novel strains contains key photosynthesis genes encoding the light-harvesting protein beta and alpha subunits (*pufBA*) and reaction centre L, M and H subunits (*pufLM* and *puhA*). Genes coding for bacteriochlorophyll (*bchIDOCXYZGPFNBHLM* and *acsF*), carotenoid (*crtIBCDEF*), photosynthesis repressor proteins (*ppsR1* and *ppsR2*) and bacteriophytochrome (*bphP*) are also present (Fig. 5).

The arrangement of genes in the PGC of 58S1^T^ and S23321 is similar to that in *B. betae* PL7HG1^T^ [4] isolated from a tumour on the roots of sugar beet [3], *B. amphicarpaeae* 39S1MB^T^ [37] isolated from a root nodule of soybean [2] and to *Rhodopseudomonas palustris* CGA009 [38]. In contrast, the arrangement of the genes in the PGC of strains 58S1^T^ and S23321 differs from *B. oligotrophicum* S58^T^ [39], a Nod factor-independent symbiont of the semi-aquatic legume, *Aeschynomene indica* (Fig. 5).

**Fig. 6.**
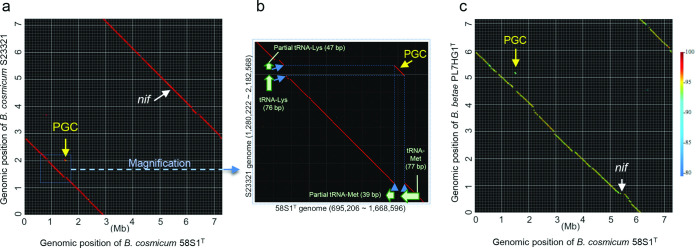
Harr plot analysis of strain 58S1^T^ vs S23321 (panels a and b) and 58S1^T^ vs *Bradyrhizobium betae* PL7HG1^T^ (panel c). Panel b represents magnification of the region around the photosynthesis gene cluster (PGC) in panel a. Green arrows (panel b) indicate the existence of tRNA genes and their partial tandem duplications adjacent to the PGC. *nif*, Indicates the position of the nitrogen fixation gene cluster (panels a and c).

Harr plot analysis [40] was employed to further characterize the genomic structure of strains 58S1^T^, S23321 and *B. betae* PL7HG1^T^ using GenomeMatcher software [35]. The results, based on comparisons with 58S1^T^, reveal colinearity of genomes (i.e. similar genes in two strains are in the same relative positions in their genomes) except for the PGC regions which are markedly nonlinear (Fig. 6a, c). Although the IslandViewer 4 [41] suite of programs did not predict the PGCs of strains 58S1^T^ and S23321 as conventional genomic islands (GIs; Fig. 4a, b), we detected a tRNA gene and its partial tandem duplication at the boundaries of the PGC region in both of these strains (Figs 5 and 6b) which may be the hallmark of flexible GIs [42, 43]. Flexible GIs of this type are typically acquired by non-homologous recombination in preferential insertion sites, such as tRNAs, and may leave behind telltale partial direct repeats [44].

It is noteworthy that we also detected a tRNA gene but not its partial repeat in the border region of the PGC of *B. betae* PL7HG1^T^ and *B. amphicarpaeae* 39S1MB^T^ [37] (Fig. 5). Collectively these observations suggest that in some species of the genus *Bradyrhizobium *, the PGC region may act as a mobile genetic element with potential for the horizontal transfer of photosynthesis genes. Comparison of the G+C contents of genomes with PGCs of strains 58S1^T^, S23321, *B. betae* PL7HG1^T^ and *B. amphicarpaeae* 39S1MB^T^ did not reveal significant differences, suggesting that any horizontal transfer of the PGC region may have occurred between closely related bacterial species or alternatively was a distant evolutionary event [45].

## Phenotypic characterization

Novel strains 58S1^T^ and S23321 produce colonies that are circular, convex, beige, translucent and <1 mm diameter after 7 days growth on yeast extract–mannitol (YEM) agar medium [6] at 28 °C. Bacterial cells are Gram-stain-negative based on the KOH method of Buck [46]. They produce an alkaline reaction on YEM agar after 21 days growth at 28°C that is typical of the genus *Bradyrhizobium *. Strains 58S1^T^ and S23321 did not produce pink-pigmented colonies on modified HM agar medium [39] after 7–14 days at 28°C under fluorescent and incandescent or natural daylight (14 h light, 10 h dark) in contrast to photosynthetic reference strains, *B* *oligotrophicum* . S58^T^, *B. denitrificans* IFAM1005^T^ and *Bradyrhizobium* sp. BTAi1. Cell morphology was investigated using transmission electron microscopy as described previously [5, 12]. Cells of 58S1^T^ and S23321 [5] are rod-shaped with sub-polar and lateral flagella. Cells of the type strain (Fig. S9) have an average cell size of 0.86×1.64 µm, which is consistent with the characteristics of the genus *Bradyrhizobium* [47].

Analysis of fatty acids was done using the Sherlock Microbial Identification System (midi) version 6.0 and the rtsba6 database as described previously [12]. Table S3 shows that novel strain 58S1^T^ exhibited a fatty acid profile characteristic of the genus *Bradyrhizobium* [48] with a predominance of fatty acids C_16  :  0_ and C_18 : 1_ω6*c*/C_18 : 1_ω7*c* (summed feature 8).

Multiple phenotypic tests including carbon source utilization and chemical sensitivity assays were carried out using Biolog GEN III MicroPlates according to the manufacturer’s instructions. The results (Table S4) show that strain 58S1^T^ can be differentiated from close relative, *B. betae* PL7HG1^T^ as well as from type strains of *B. cytisi *, *B. rifense *, *B. canariense *, *B. japonicum* and *B. diazoefficiens* on the basis of several of these phenotypic tests.

Plant tests were carried out using modified Leonard jars as described previously [2, 5, 6] with *B. diazoefficiens* USDA110^T^ and *B. oligotrophicum* S58^T^ as reference strains. Based on the results of these tests, strains 58S1^T^ and S23321 did not elicit nodules on roots of *Macroptilium atropurpureum* ‘Siratro’ or *Aeschynomene indica*. Further tests showed that 58S1^T^ did not elicit nodules on soybean ‘AC Orford’ or *Amphicarpaea bracteata*. The fact that strain 58S1^T^ was originally isolated from a root nodule of soybean [2] suggests that it may be an opportunist that occasionally occupies root nodules.

Based on the phylogenetic, complete genome sequence and phenotypic data presented here, we propose that strains 58S1^T^ and S23321 represent a novel species named *Bradyrhizobium cosmicum* sp. nov.

## Description of *Bradyrhizobium cosmicum* sp. nov.

*Bradyrhizobium cosmicum* (cos'mi.cum. L. neut. adj. *cosmicum*, of the world, cosmopolitan).

Cells are Gram-stain-negative, aerobic, non-spore-forming rods (approx. 0.86 µm wide and 1.64 µm long) with sub-polar and lateral flagella. Colonies on YEM agar medium are circular, convex, beige, translucent and <1 mm in diameter after 7 days at 28°C. Growth occurs at pH 5–10 (optimum, pH 7.0). Produces an alkaline reaction on YEM agar. The type strain grows at 10°C, optimal at 28°C, but no growth occurs at 37 °C. Does not produce pink-pigmented colonies on modified HM agar medium when exposed to light–dark cycles. The type strain does not grow in the presence of 1 % (w/v) NaCl. Utilizes d-mannose, d-galactose, d-fucose, l-fucose, d-mannitol, d-arabitol, l-pyroglutamic acid, l-galactonic acid lactone, d-gluconic acid, mucic acid, d-saccharic acid, methyl pyruvate, l-lactic acid, α-keto-glutaric acid, d-malic acid, l-malic acid, Tween 40, β-hydroxy-d,l-butyric acid, acetic acid, formic acid and four other carbon sources. Does not utilize d-sorbitol, d-glucose-6-PO4, d-fructose-6-PO4, d-aspartic acid, l-glutamic acid, pectin, quinic acid, citric acid, α-hydroxybutyric acid, acetoacetic acid, propionic acid and 34 other carbon sources. Resistant to 1 % sodium lactate, troleandomycin, lincomycin, nalidixic acid and four other chemical compounds. Susceptible to nalidixic acid, potassium tellurite, aztreonam and six other chemical compounds.

Predominant fatty acids are C_16 : 0_ and C_18 : 1_ω6*c*/C_18 : 1_ω7*c* (summed feature 8). Does not elicit root nodules on *Glycine max*, *Macroptilium atropurpureum*, *Amphicarpaea bracteata* or *Aeschynomene indica*.

The type strain, 58S1^T^ (=LMG 31545^T^=HAMBI 3725^T^), was isolated from a root nodule of a soybean plant that was inoculated with root-zone soil of *Amphicarpaea bracteata* (Hog peanut) growing in Canada. The type strain contains key photosystem and nitrogen-fixation genes but not nodulation or type III secretion system genes. The DNA G+C content of the type strain is 64.3 mol% and the genome size is 7.30 Mbp. GenBank/EMBL/DDBJ accession numbers for the complete genome and the 16S rRNA, *atpD*, *glnII*, *recA*, *gyrB*, *rpoB* and *nifH* gene sequences of the type strain are CP041656, KP768789, KP768557, KP768615, KF615104, KP768731, KP768673 and CP041656, respectively.

## Supplementary Data

Supplementary material 1
