# Supplementary material for: Strains of Bradyrhizobium cosmicum sp. nov., isolated from contrasting habitats in Japan and Canada possess photosynthesis gene clusters with the hallmark of genomic islands
Source: Int J Syst Evol Microbiol. 2020 Aug 17;70(9):5063–74. doi: 10.1099/ijsem.0.004380 (PMC7656271; doi:10.1099/ijsem.0.004380)
Supplement: Supplementary material 1 [file ijsem-70-5063-s001.pdf]

Supplementary Table S1. GenBank nucleotide accession numbers for *Bradyrhizobium cosmicum* sp. nov. strains 58S1^T and S23321 and reference taxa.

Strain	*atpD*	*glnII*	*gyrB*	*recA*	*rpoB*	*16S rRNA*	*nifH*
***Bradyrhizobium cosmicum* 58S1^T**	KP768557	KP768615	KP768731	KF615104	KP768673	KP768789	CP041656
***Bradyrhizobium cosmicum* S23321**	AP012279	AP012279	AP012279	AP012279	AP012279	AP012279	AP012279
B. algeriense RST89^T	PYCM01000338	PYCM01000763	PYCM01000687	PYCM01000643	PYCM01000653	PYCM01000149	FJ348678
B. americanum CMVU44^T	-	KX012942	NA	KC247141	NA	KU991833	KC247135
B. amphicarpaeae 39S1MB^T	KP768547	KP768605	KF615002	KP768721	KP768663	KP768779	KF615664
B. arachidis CCBAU 051107 ^{T}	KF962683	KF962689	KF962693	KF962707	JX437682	HM107167	KF962700
B. betae PL7HG1^T	FM253129	AB353733	FM253217	AB353734	FM253260	NR_029104	NA
B. brasilense UFLA03-321^T	KF452730	MPVQ01000074	KF452827	KT793142	MPVQ01000073	MPVQ01000039	MPVQ01000047
B. cajani AMBPC1010^T	NA	KY349442	NA	KY349440	NA	KY349447	NA
B. canariense BTA-1^T	FM253135	AY386765	FM253220	AY591553	FM253263	AJ558025	EU818926
B. centrolobii BR 10245^T	LUUB01000107	LUUB01000131	LUUB01000063	LUUB01000078	LUUB01000064	LUUB01000105	LUUB01000073
B. centrosematis A9^T	-	KX012940	NA	KC247145	NA	KC247115	KC247139
B. cytisi CTAW11^T	JN186289	JN186291	JN186292	JN186293	JN186288	EU561065	GU001618
B. daqingense CCBAU 15774^T	HQ231289	KF962690	KF962694	KF962708	JX437676	KJ184551	KF962701
B. denitrificans IFAM 1005^T	FM253153	HM047121	FM253239	EU665419	FM253282	NR_041827	HM047125
B. diazoefficiense USDA 110 ^{T}	BA000040	BA000040	BA000040	BA000040	BA000040	BA000040	AB000040
B. elkanii USDA 76^T	AY386758	AY599117	AM418800	AY591568	EF190188	HQ233240	AB094963
B. embrapense CNPSo 2833^T	LFIP02000012	LFIP0200001	LFIP0200010	LFIP0200009	LFIP0200004	AY904773	KP234518
B. erythrophlei CCBAU 53325^T	NA	KF114693	-	KF114669	-	KF1144645	KF114598
B. ferriligni CCBAU 51502^T	NA	KJ818099	-	KJ818112	-	KX683400	KJ818108
B. forestalis INPA54B^T	PGVG01000048	KF452867	PGVG01000049	PGVG01000004	PGVG01000026	PGVG01000009	PGVG00000000
B. frederickii CNPSo 3426^T	SPQS01000024	SPQS01000012	SPQS01000005	SPQS01000004	SPQS01000016	SPQS01000017	SPQS01000036
B. ganzhouense RITF806^T	-	JX277110	NA	JX277144	NA	JQ796661	JX292065
B. guangdongense CCBAU 51649^T	CP030051	CP030051	CP030051	CP030051	CP030051	CP030051	KC509130
B. guangxiense CCBAU 53363^T	CP022219	CP022219	CP022219	CP022219	CP022219	CP022219	KC509140
B. guangzhouense CCBAU 51670^T	CP030053	CP030053	CP030053	CP030053	CP030053	CP030053	SPQS01000036
B. huanghuaihaiense CCBAU 23303^T	HQ231682	KF962691	KF962695	KF962709	JX437679	HQ231463	KF962702
B. icense LMTR 13^T	KF896192	KF896175	KF896201	JX943615	CP016428	KF896156	KF896161
B. ingae BR 10250^T	-	KF927067	-	KF927061	-	KF927043	KF927085*
B. iriomotense EK05^T	AB300994	AB300995	HQ873308	AB300996	HQ587646	AB300992	AB300998*
B. ivorense CI-1B^T	CAADFC020000004	CAADFC020000018	CAADFC020000001	CAADFC020000009	CAADFC020000027	CAADFC020000004	CAADFC020000008
B. japonicum USDA 6^T	AM168320	HQ587875	AM418801	AM168341	AY242830	AB510002	HM047126
B. jicamae PAC68^T	FJ428211	FJ428204	HQ873309	HQ587415	HQ587647	AY624134	HM047127

Strain	*atpD*	*glnII*	*gyrB*	*recA*	*rpoB*	*16S rRNA*	*nifH*
B. kavangense 14-3[T]	KY753592	KM378446	KX661397	KM378399	KM378311	KP899562	KM378254[*]
B. lablabi CCBAU 23086[T]	GU433473	GU433498	KF962696	KF962710	JX437677	GU433448	KF962703
B. liaoningense 2281[T]	FM253137	AY386775	FM253223	AY591564	FM253266	AJ250813	EU818925
B. lupini USDA 3051[T]	-	KM114862	-	KM114866	NA	KM114861	NA
B. macuxiense BR 10303[T]	LNCU01000024	LNCU01000062	LNCU01000041	LNCU01000014	LNCU01000011	LNCU01000022	LNCU00000000
B. manausense BR 3351[T]	LJYG01000004	LJYG01000112	LJYG01000105	LJYG01000054	LJYG01000045	HQ641226	LJYG00000000
B. mercantei SEMIA 6399[T]	MKFI01000006	KX690621	KX690617	KX690615	MKFI01000001	FJ025102	KX690625
B. namibiense 5-10[T]	KX661387	KM378440	KX661393	KM378377	KM378306	KX661401	KM378249[*]
B. nanningense CCBAU 53390[T]	LBJC01000078	LBJC01000005	LBJC01000024	LBJC01000019	LBJC01000081	LBJC01000082	LBJC01000002
B. neotropical BR 10247[T]	LSEF01000046	KJ661700	LSEF01000100	KF785992	KF983829	LSEF01000032	LSEF00000000
B. niftali CNPSo 3448[T]	SPQT01000036	SPQT01000002	SPQT01000008	SPQT01000004	SPQT01000028	SPQT01000015	SPQT01000005
B. nitroreducens TSA1[T]	LFJC01000003	LFJC01000003	LFJC01000003	LFJC01000003	LFJC01000003	LFJC01000003	AB542336
B. oligotrophicum S58[T]	JQ619232	JQ619233	KF962697	JQ619231	KF962713	JQ619230	AP012603
B. ottawaense OO99[T]	HQ455212	HQ587750	HQ873179	HQ587287	HQ587518	JN186270	JN186287
B. pachyrhizi PAC48[T]	FJ428208	FJ428201	HQ873310	HQ587416	HQ587648	AY624135	HM047124
B. paxllaeri LMTR 21[T]	CP042968	CP042968	CP042968	CP042968	CP042968	AY923031	DQ085619
B. retamae Ro19 [T]	KC247101	KC247108	KF962698	KF962711	KF962714	LLYA01000002	KF962704
B. rifense CTAW71[T]	GU001617	KF962692	KF962699	KF962712	KF962715	EU561074	GU001627
B. ripae WR4[T]	NA	MF593086	-	MF593090	-	MF593082	NA
B. sacchari BR 10280[T]	KX065107	KX065099	LWIG01000001	KX065095	LWIG01000014	KF113091	LWIG00000000
B. shewense ERR11[T]	FMAI01000019	FMAI01000022	FMAI01000013	FMAI01000022	FMAI01000007	FMAI01000022	FMAI00000000
B. stylosanthis BR 446[T]	LVEM01000002	LVEM01000001	LVEM01000002	LVEM01000001	LVEM01000001	LVEM01000016	KU724157
B. subterraneum 58 2-1[T]	-	KM378484	-	KM378397	-	KP308152	KM378289[*]
B. symbiodeficiens 85S1MB[T]	KP768551	KP768609	KP768725	KF615036	KP768667	KP768783	NA
B. tropiciagri CNPSo 1112[T]	LFLZ01000050	LFLZ01000067	LFLZ01000066	LFLZ01000039	LFLZ01000008	LFLZ01000084	HQ259540
B. uaiense UFLA03-164[T]	VKHP01000008	VKHP01000010	VKHP01000007	VKHP01000115	VKHP01000007	VKHP01000252	VKHP01000057
B. valentinum LmjM3[T]	LLXX01000203	LLXX01000118	LLXX01000044	LLXX01000017	LLXX01000029	JX514883	KF806461
B. vignae 7-2[T]	RDQF01000079	RDQF01000005	RDQF01000001	RDQF01000045	RDQF01000055	RDQF01000051	RDQF01000014
B. viridifuturi SEMIA 690[T]	LGTB01000039	LGTB01000012	LGTB01000021	LGTB01000025	LGTB01000001	FJ025107	KR149137
B. yuanmingense CCBAU 10071[T]	FM253140	AY386780	FM253226	AM168343	FM253269	AB509380	EU818927
B. zhanjiangense CCBAU 51778[T]	CP022221	CP022221	CP022221	CP022221	CP022221	CP022221	RDQF01000014

NA Sequences not available in public databases.

- Sequence not used in phylogenetic analyses of five housekeeping genes.

[*] Sequences used only for % sequence similarity estimates (Table 1) and not for phylogenetic analyses.

Table S2. Sequence similarities (%) for five concatenated housekeeping genes, 16S rRNA and *nif*H genes of *Bradyrhizobium cosmicum* sp. nov. strains 58S1^T (1) and S23321 (2) versus reference taxa.

Sequence similarity (%) (coverage if not 100%*)													
**Strain**	**Five genes† (2679 bp)**		**16S rRNA (1422 bp)**		***nif*H (540bp)**		**Strain**	**Five genes† (2679 bp)**		**16S rRNA (1422 bp)**		***nif*H (540bp)**	
	**1**	**2**	**1**	**2**	**1**	**2**		**1**	**2**	**1**	**2**	**1**	**2**
***Bradyrhizobium cosmicum* 58S1^T**	-	98.7	-	100.0‡		98.7	*Bradyrhizobium jicamae* PAC68^T	90.1	90.1	97.4	96.9	88.7	88.5
***Bradyrhizobium cosmicum* S23321**	98.7	-	100.0‡	-	98.7	-	*Bradyrhizobium kavangense* 14-3^T	93.4	93.2	98.1 (95)	98.1 (95)	85.0 (51)	84.7 (51)
Bradyrhizobium. algeriense RST89^T	89.2	89.2	97.8 (92)	97.6 (92)	89.8	89.8	*Bradyrhizobium lablabi* CCBAU 23086^T	90.1	90.0	97.2	97.0	90.6	90.6
Bradyrhizobium americanum CMVU44^T	NA	NA	98.4	98.4	86.1	85.7	*Bradyrhizobium liaoningense* 2281^T	94.1	93.8	98.7	98.7	83.9	83.7
Bradyrhizobium amphicarpaeae 39S1MB^T	94.4	94.4	99.5	99.4	91.9	91.7	*Bradyrhizobium lupini* USDA 3051^T	NA	NA	98.7	98.7	NA	NA
Bradyrhizobium arachidis CCBAU 051107^T	93.8	93.6	98.7	98.7	86.3	86.3	*Bradyrhizobium macuxiense* BR 10303^T	91.0	91.0	97.1	97.0	85.7	85.7
Bradyrhizobium. betae PL7HG1^T	97.2	97.2	99.6	99.6	NA	NA	*Bradyrhizobium manausense* BR 3351^T	93.2	93.0	98.5	98.5	87.6	87.0
Bradyrhizobium brasilense UFLA 03-321^T	90.8	90.7	97.0	97.0	85.4	85.9	*Bradyrhizobium mercantei* SEMIA 6399^T	90.7	90.7	97.3	97.3	85.4	85.4
Bradyrhizobium cajani AMBPC1010^T	NA	NA	98.4	98.4	NA	NA	*Bradyrhizobium namibiense* 5-10^T	89.5	89.4	97.3	97.3	90.1 (51)	89.8 (51)
Bradyrhizobium canariense BTA-1^T	94.4	94.3	98.9	98.9	89.6	89.1	*Bradyrhizobium nanningense* CCBAU 53390^T	93.2	93.3	99.0	99.0	84.8	84.6
Bradyrhizobium centrolobii BR 10245^T	92.8	94.6	96.7 (94)	96.2 (94)	88.7	88.5	*Bradyrhizobium neotropicale* BR 10247^T	92.8	92.7	96.9	96.9	90.2	89.8
Bradyrhizobium centrosematis A9^T	NA	NA	99.0	99.0	84.4	83.7	*Bradyrhizobium niftali* CNPSo 3448^T	95.0	95.2	98.8	98.8	87.0	87.0
Bradyrhizobium cytisi CTAW11^T	94.1	94.0	99.1	99.1	89.6	89.1	*Bradyrhizobium nitroreducens* TSA1^T	94.4	94.2	99.2‡	99.2‡	91.9	91.7
Bradyrhizobium daqingense CCBAU 15774^T	92.9	92.8	98.4	98.4	83.7	83.5	*Bradyrhizobium oligotrophicum* S58^T	88.9	88.8	98.4	98.4	91.7	91.7
Bradyrhizobium denitrificans IFAM 1005 ^{T}	89.5	89.4	98.3	98.3	90.9	90.7	*Bradyrhizobium ottawaense* OO99^T	94.0	93.8	98.9	98.9	83.9	83.7
Bradyrhizobium diazoefficiens USDA 110^T	95.3	95.3	98.8	98.8	83.9	83.7	*Bradyrhizobium pachyrhizi* PAC48^T	90.6	90.5	97.0	96.9	86.3	86.3
Bradyrhizobium elkanii USDA 76^T	91.2	91.1	97.0	96.8	87.6	87.6	*Bradyrhizobium paxllaeri* LMTR21^T	89.8	89.7	97.3	97.2	90.2	90.2
Bradyrhizobium embrapense CNPSo 2833^T	90.7	90.5	97.3	97.1	85.9	86.5	*Bradyrhizobium retamae* Ro19 ^{T}	88.9	88.9	97.5	97.5	89.8	89.8

Sequence similarity (%) (coverage if not 100%*)

Strain	Five genes† (2679 bp)		16S rRNA (1422 bp)		*nif*H (540bp)		Strain	Five genes† (2679 bp)		16S rRNA (1422 bp)		*nif*H (540bp)	
	1	2	1	2	1	2		1	2	1	2	1	2
Bradyrhizobium erythrophlei CCBAU 53325^{T}	NA	NA	97.7 (95)	97.6 (95)	85.7	85.7	*Bradyrhizobium rifense* CTAW71^{T}	94.9	94.8	98.9	98.9	89.1	88.5
Bradyrhizobium ferriligni CCBAU 51502^{T}	NA	NA	97.3 (99)	96.9 (99)	88.0	88.0	*Bradyrhizobium ripae* WR4^{T}	NA	NA	97.0	96.9	NA	NA
Bradyrhizobium forestalis INPA54B^{T}	92.3	92.3	98.7	98.7	86.9	86.8	*Bradyrhizobium sacchari* BR 10280^{T}	93.1	92.7	98.6	98.6	92.6	92.2
Bradyrhizobium frederickii CNPSo 3426^{T}	93.4	93.1	99.1	99.1	86.9	86.8	*Bradyrhizobium shewense* ERR11^{T}	94.2	94.2	98.8	98.8	87.0	87.0
Bradyrhizobium ganzhouense RITF806^{T}	NA	NA	98.7 (97)	98.7 (97)	89.1	88.7	*Bradyrhizobium stylosanthis* BR 446^{T}	94.4	94.2	98.8 (93)	98.8 (93)	87.0	98.7
Bradyrhizobium guangdongense CCBAU 51649^{T}	93.6	93.5	99.1 (90)	99.1 (90)	86.5	82.3	*Bradyrhizobium subterraneum* 58 2-1^{T}	NA	NA	98.9 (94)	98.9 (94)	86.1 (51)	85.8 (51)
Bradyrhizobium guangxiense CCBAU 53363^{T}	93.4	93.2	99.4 (89)	99.4 (89)	93.0	92.6	*Bradyrhizobium symbiodeficiens* 85S1MB^{T}	94.4	94.4	99.1	99.0	NA	NA
Bradyrhizobium guangzhouense CCBAU 51670^{T}	93.6	93.7	98.6	98.6	86.5	86.3	*Bradyrhizobium tropiciagri* CNPSo 1112^{T}	91.4	91.3	97.0	96.9	86.3	86.8
Bradyrhizobium huanghuaihaiense CCBAU 23303^{T}	94.2	94.0	98.9	98.9	83.9	83.7	*Bradyrhizobium uaiense* UFLA03-164^{T}	90.9	90.8	97.5‡	97.5‡	86.1	86.7
Bradyrhizobium icense LMTR 13^{T}	89.1	89.1	97.3	97.2	90.4	90.4	*Bradyrhizobium valentinum* LmjM3^{T}	89.4	89.3	97.2	97.1	89.6	89.6
Bradyrhizobium ingae BR 10250^{T}	NA	NA	98.2 (96)	98.2 (96)	85.9 (38)	86.3 (38)	*Bradyrhizobium vignae* 7-2^{T}	92.9	92.5	98.8 (92)	98.8 (92)	87.0	87.0
Bradyrhizobium iriomotense EK05^{T}	92.0	92.2	98.3	98.3	84.8 (51)	84.7 (51)	*Bradyrhizobium viridifuturi* SEMIA 690^{T}	91.2	91.0	97.3	97.3	85.9	86.5
Bradyrhizobium ivorense CI-1B^{T}	90.6	90.6	96.9	97.0	86.7	86.7	*Bradyrhizobium yuanmingense* CCBAU 10071^{T}	92.8	92.9	98.7	98.7	87.2	87.2
Bradyrhizobium japonicum USDA 6^{T}	94.8	95.0	98.8	98.8	83.9	83.7	*Bradyrhizobium zhanjiangense* CCBAU 51778^{T}	92.9	92.7	98.1	98.1	86.7	86.7

NA Sequence not available in public databases.

* Short sequences (*nif*H short sequences were not used in the phylogenetic analysis shown in Fig. 2).

† five concatenated genes: *atp*D-*gln*II-*gyr*B-*rec*A-*rpo*B.

‡ 16S rRNA gene similarities calculated using sequences from GenBank as reference sequences not available in EzBioCloud database (Yoon SH, Ha SM, Kwon S, Lim J, Kim Y ***et al.*** *Int J Syst Evol Microbiol* 2017; 67:1613–1617).

Supplementary Table S3. Fatty acid patterns of *Bradyrhizobium* strains: **1**. *B. cosmicum* sp. nov. 58S1^T, **2**. *B. betae* PL7HG1^T, **3**. *B. cytisi* CTAW11^T, **4**. *B. rifense* CTAW71^T, **5**. *B. japonicum* USDA6^T and **6**. *B. diazoefficiense* USDA110^T.

**Fatty Acid**	**1**	**2**	**3**	**4**	**5***	**6†**
12:0	0.7	2.2	1.1	1.0	-	-
14:0	-	1.2	0.5	-	-	-
15:1 ω5c	-	-	0.6	-	-	-
16:0	8.8	11.4	8.8	7.8	13.1	14.1
16:1 ω11c	-	-	0.5	-	-	-
16:1 ω5c	2.6	3.9	6.1	8.6	3.6	-
17:1 ω8c	1.9	0.6	1.2	0.7	-	-
17:1 ω6c	0.8	0.4	0.8	-	-	-
17:0	0.8	0.4	0.5	-	-	-
18:0	1.5	7.5	2.7	1.9	0.8	-
18:0 iso	-	-	1.9	-	-	-
C18:1 ω7c 11-methyl	3.6	1.1	2.1	-	6.7	-
19:0 cyclo ω8c			1.8	2.6	-	-
20:0	-	-	0.5	2.5	-	-
20:0 iso	-	0.8	-	-	-	-
Summed feature 2§	-	1.2	-	-	-	-
Summed feature 3§	1.6	3.0	1.4	3.3	1.1	-
Summed feature 5§	-	1.2	-	-	-	-
Summed feature 8§	77.0	59.0	67.5	70.9	74.8	85.9

- Not detected.

* Data from: Yu X, Cloutier S, Tambong J, Bromfield ESP. *Int J Syst Evol Microbiol* 2014; 64: 3202–3207.

† Data from: Delamuta JR, Ribeiro RA, Ormeño-Orrillo E, Melo IS, Martínez-Romero E *et al. Int J Syst Evol Microbiol* 2013; 63: 3342-3351.

§ Summed features represent groups of fatty acids that were not separated by GLC with the MIDI system. Summed feature 2, 12:0 aldehyde/?; Summed feature 3, 16:1 ω6c/16:1 ω7c; Summed feature 5, 18:0 ante/18:2 ω6,9c; Summed feature 8, 18:1 ω6c/18:1 ω7c.

Supplementary Table S4. Phenotypic characteristics of **1**. *B.cosmicum* sp. nov. 58S1^T, **2**. *B. betae* PL7HG1^T, **3.** *B. cytisi* CTAW11^T, **4.** *B. rifense* CTAW71^T, **5.** *B. canariense* BTA-1^T, **6.** *B. japonicum* USDA 6^T and **7**. *B. diazoefficiens* USDA 110^T.

**Characteristic**	**1**	**2**	**3**	**4**	**5**	**6**[a]	**7**[a]	**Characteristic**	**1**	**2**	**3**	**4**	**5**	**6**[a]	**7**[a]
C-source utilization (Biolog)[b]															
Dextrin	-	-	-	-	-	-	-	Glycyl-L-Proline	-	-	-	-	-	-	-
D-Maltose	-	-	-	-	-	-	-	L-Alanine	-	-	-	-	-	-	-
D-Trehalose	-	-	-	-	-	-	-	L-Arginine	-	-	-	-	-	-	-
D-Cellobiose	-	-	-	-	-	-	-	L-Aspartic Acid	-	-	-	-	-	-	-
Gentiobiose	-	-	-	-	-	-	-	L-Glutamic Acid	-	±	-	-	-	-	±
Sucrose	-	-	-	-	-	-	-	L-Histidine	-	-	-	-	-	-	-
D-Turanose	-	-	-	-	-	-	-	L-Pyroglutamic Acid	+	+	-	-	-	-	+
Stachyose	-	-	-	-	-	-	-	L-Serine	-	-	-	-	-	-	-
D-Raffinose	-	-	-	-	-	-	-	Pectin	-	-	±	-	-	-	-
α-D-Lactose	-	-	-	-	-	-	-	D-Galacturonic Acid	+	+	±	+	±	+	+
D-Melibiose	-	-	-	-	-	-	-	L-Galactonic Acid Lactone	+	+	±	+	±	-	+
β-Methyl-DGlucoside	-	-	-	-	-	-	-	D-Gluconic Acid	+	+	±	-	±	-	+
D-Salicin	-	-	-	-	-	-	-	D-Glucuronic Acid	+	+	±	+	±	+	+
N-Acetyl-DGlucosamine	-	-	-	-	-	-	-	Glucuronamide	+	+	+	+	+	+	+
N-Acetyl-β-DMannosamine	-	-	-	-	-	-	-	Mucic Acid	±	+	-	±	+	+	+
N-Acetyl-DGalactosamine	-	-	-	-	-	-	-	Quinic Acid	-	±	-	-	+	±	±
N-Acetyl Neuraminic Acid	-	-	-	-	-	-	-	D-Saccharic Acid	±	+	-	-	+	+	+
α-D-Glucose	-	-	-	-	-	-	-	p-HydroxyPhenylacetic Acid	-	-	-	-	-	-	-
D-Mannose	+	±	-	-	±	-	-	Methyl Pyruvate	+	+	-	-	-	+	+
D-Fructose	-	-	-	-	-	-	-	D-Lactic Acid Methyl Ester	-	-	-	-	-	-	-
D-Galactose	+	±	-	-	±	-	±	L-Lactic Acid	±	+	-	-	-	+	+
3-Methyl Glucose	-	-	-	-	-	-	-	Citric Acid	-	-	-	-	±	-	-
D-Fucose	+	+	-	-	±	+	+	α-Keto-Glutaric Acid	+	+	-	-	±	-	+
L-Fucose	±	±	-	-	±	-	±	D-Malic Acid	+	+	-	-	+	+	+
L-Rhamnose	-	-	-	-	-	-	-	L-Malic Acid	±	+	-	-	±	+	+
Inosine	-	-	-	-	-	-	-	Bromo-Succinic Acid	-	±	-	-	-	-	+
D-Sorbitol	-	-	±	±	+	-	-	Tween 40	±	±	±	-	±	-	±
D-Mannitol	+	-	±	+	+	-	-	γ-Amino-Butyric Acid	-	-	-	-	-	-	-
D-Arabitol	+	-	±	+	+	-	-	α-HydroxyButyric Acid	-	-	-	-	-	-	±
myo-Inositol	-	-	±	±	±	-	-	β-Hydroxy-D,LButyric Acid	+	+	-	-	-	+	+
Glycerol	+	+	±	±	+	+	+	α-Keto-Butyric Acid	-	-	-	-	-	-	-
D-Glucose- 6-PO4	-	-	±	+	±	-	-	Acetoacetic Acid	-	-	-	±	±	-	-
D-Fructose- 6-PO4	-	±	+	+	±	-	-	Propionic Acid	-	-	-	-	-	-	+
D-Aspartic Acid	-	-	±	±	-	-	-	Acetic Acid	+	+	-	-	±	-	+
D-Serine	-	-	-	-	-	-	-	Formic Acid	+	+	-	-	+	+	+
Gelatin	-	-	-	-	-	-	-								
Chemical Sensitivity (Biolog)[b]															
1% Sodium Lactate	±	±	±	-	-	+	±	Vancomycin	-	±	±	±	±	+	-
Fusidic Acid	-	-	±	-	-	-	-	Tetrazolium Violet	±	±	+	+	+	+	±
D-Serine	-	-	-	-	-	-	-	Tetrazolium Blue	+	+	+	+	+	+	+
Troleandomycin	±	+	±	+	±	-	+	Nalidixic Acid	±	±	±	-	±	+	±
Rifamycin SV	±	±	±	+	±	+	+	Lithium Chloride	-	-	-	-	-	-	-
Minocycline	±	±	±	+	±	+	+	Potassium Tellurite	-	-	-	-	-	-	±
Lincomycin	±	±	±	-	±	+	±	Aztreonam	-	±	±	±	±	+	-
Guanidine HCl	-	-	-	-	-	-	-	Sodium Butyrate	-	-	-	-	-	-	-
Niaproof 4	-	-	-	-	-	-	-	Sodium Bromate	-	-	-	-	-	-	-
Growth on YEM agar medium:															
10 °C	±	±	±	-	-	ND	ND								
37 °C	-	-	-	-	-	-	-								
pH 5	+	+	+	+	+	+[c]	+								
pH 10	+	±	±	-	-	ND	+								
1% NaCl	-	+	-	-	-	+[d]	-								
Acid/alkali production (pH)[e]	8.2	ND	ND	ND	ND	7.8	ND								

[a] Data from Bromfield ESP, Cloutier S and Nguyen HDT. *Int J Syst Evol Microbiol* 2020; 70: 442-449.

[b] BIOLOG GEN III MicroPlates (120 hours incubation at 28 ºC): +, Positive; ±, weak; −, negative. ND, not determined.
Values are based on three replicates.

[c] Data from Delamuta JR, Ribeiro RA, Ormeño-Orrillo E, Melo IS, Martínez-Romero E, *et al. Int J Syst Evol Microbiol* 2013; 63: 3342–3351.

[d] Data from Rivas R, Willems A, Palomo JL, Garcı´a-Benavides P, Mateos PF, ***et al.*** *Int J Syst Evol Microbiol* 2004; 54: 1271–1275.

[e] Acid/alkali production after 21 days at 28 C; uninoculated control, pH 7.1.

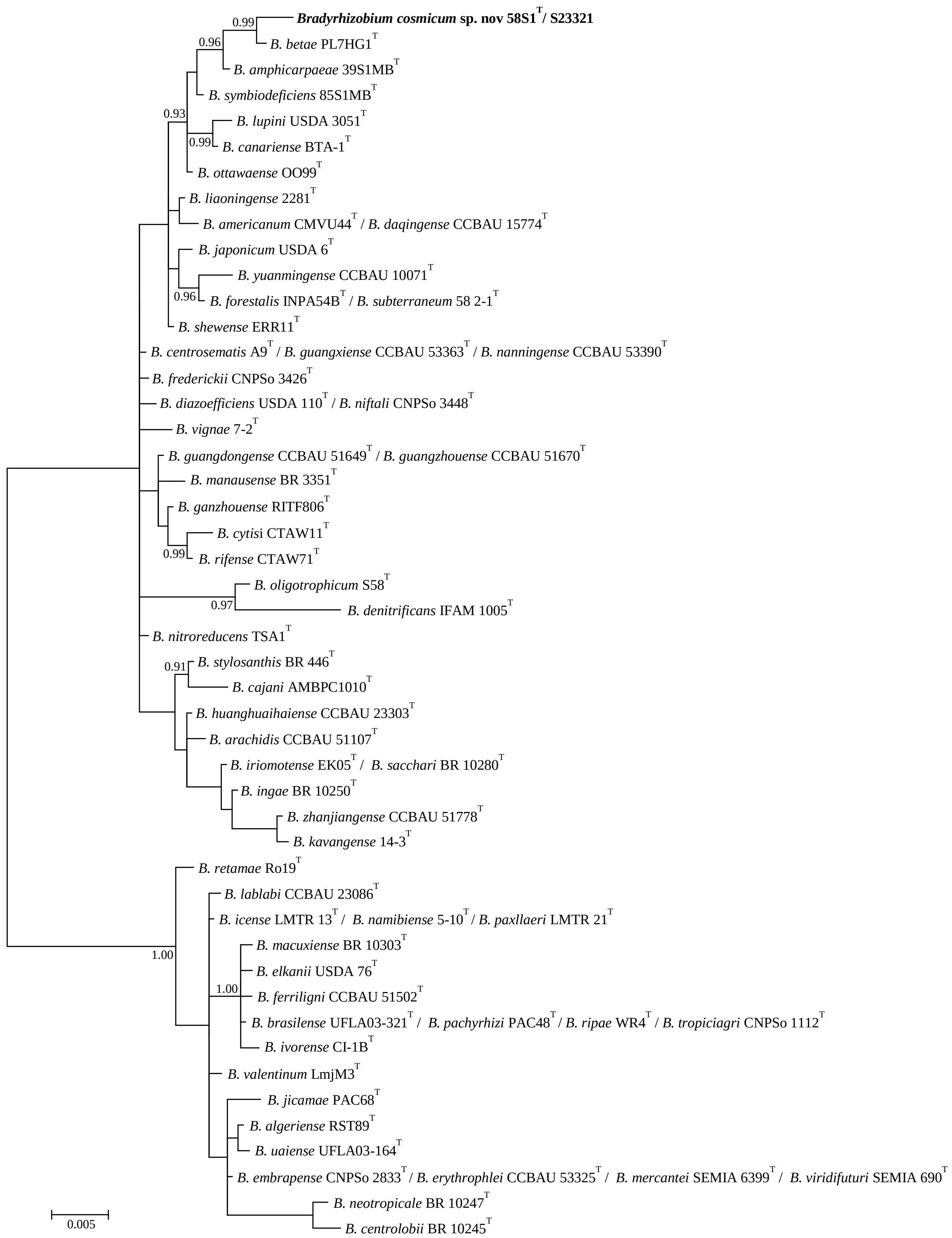

0.99
Bradyrhizobium cosmicum sp. nov 58S1T / S23321
0.96
B. betae PL7HG1T
B. amphicarpaeae 39S1MBT
B. symbiodeficiens 85S1MBT
0.93
B. lupini USDA 3051T
0.99
B. canariense BTA-1T
B. ottawaense OO99T
B. liaoningense 2281T
B. americanum CMVU44T / B. daqingense CCBAU 15774T
B. japonicum USDA 6T
B. yuanmingense CCBAU 10071T
0.96
B. forestalis INPA54BT / B. subterraneum 58 2-1T
B. shewense ERR11T
B. centrosematis A9T / B. guangxiense CCBAU 53363T / B. nanningense CCBAU 53390T
B. fredericki CNPSo 3426T
B. diazoefficiens USDA 110T / B. niftali CNPSo 3448T
B. vignae 7-2T
B. guangdongense CCBAU 51649T / B. guangzhouense CCBAU 51670T
B. manausense BR 3351T
B. ganzhouense RITF806T
B. cytisi CTAW11T
0.99
B. rifense CTAW71T
B. oligotrophicum S58T
0.97
B. denitrificans IFAM 1005T
B. nitroreducens TSA1T
0.91
B. stylosanthis BR 446T
B. cajani AMBPC1010T
B. huanghuaihaense CCBAU 23303T
B. arachidis CCBAU 51107T
B. iriomotense EK05T / B. sacchari BR 10280T
B. ingae BR 10250T
B. zhanjiangense CCBAU 51778T
B. kavangense 14-3T
B. retamae Ro19T
B. lablabi CCBAU 23086T
B. icense LMTR 13T / B. namibiense 5-10T / B. paxllaeri LMTR 21T
B. macuxiense BR 10303T
B. elkanii USDA 76T
1.00
B. ferriligni CCBAU 51502T
B. brasilense UFLA03-321T / B. pachyrhizi PAC48T / B. ripae WR4T / B. tropiciagri CNPSo 1112T
B. ivorense CI-1BT
1.00
B. valentinum LmjM3T
B. jicamae PAC68T
B. algeriense RST89T
B. uaiense UFLA03-164T
B. embrapense CNPSo 2833T / B. erythrophlei CCBAU 53325T / B. mercantei SEMIA 6399T / B. viridifuturi SEMIA 690T
B. neotropicale BR 10247T
B. centrolobii BR 10245T
0.005

Supplementary Fig. S1 Bayesian phylogenetic tree of 16S rRNA gene sequences for *Bradyrhizobium cosmicum* sp. nov., and reference taxa (HKY + G + I substitution model). Only posterior probabilities ≥ 0.90 are shown. Scale bar represents expected number of substitutions per site.

To include all named species of *Bradyrhizobium* in the tree, alignment lengths of 16S rRNA gene sequences were trimmed to 1300 bp.

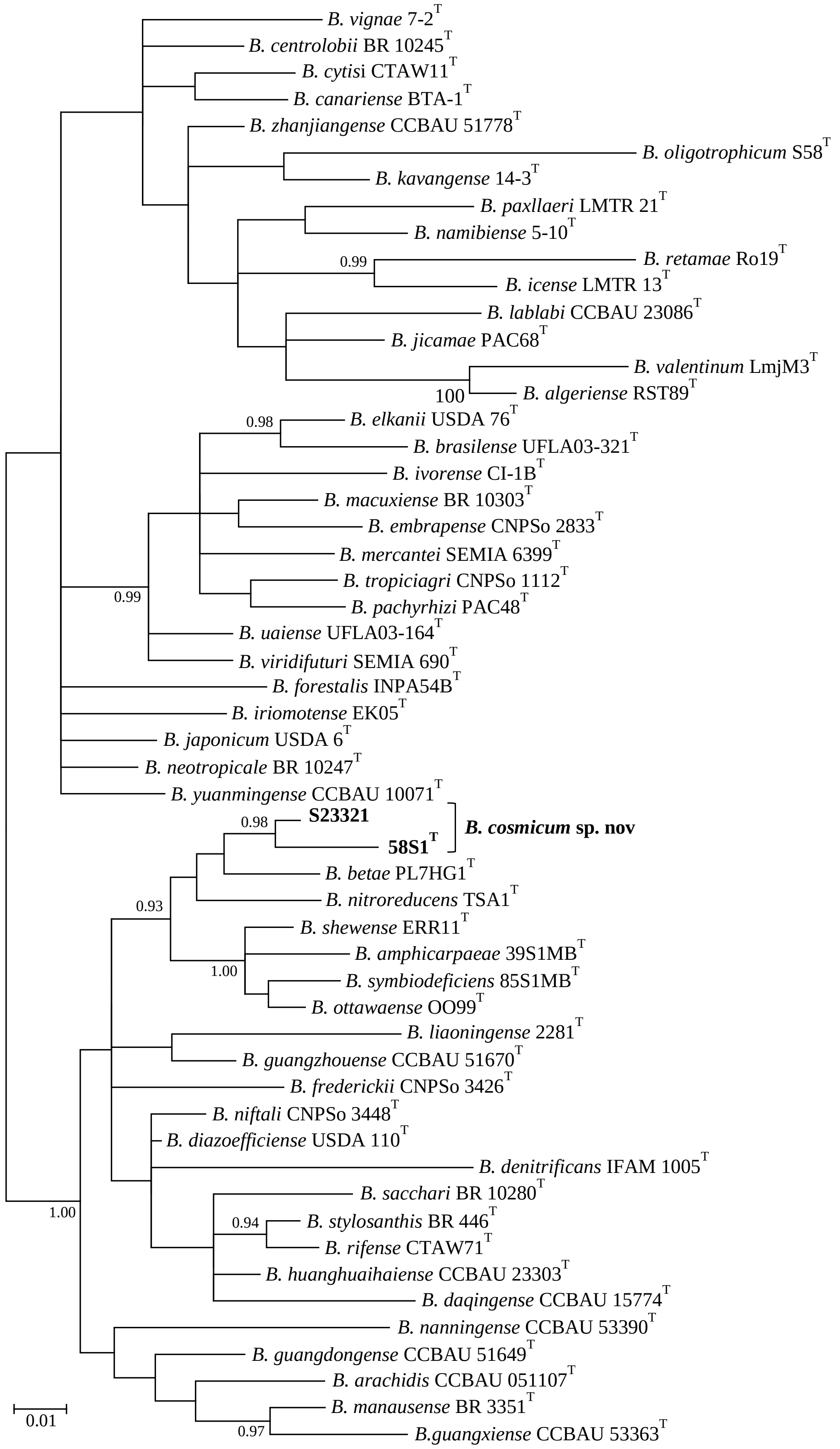

Fig. S2. Bayesian phylogenetic tree (GTR + G + I substitution model) of *atp*D housekeeping gene sequences (429 bp) for *Bradyrhizobium cosmicum* sp. nov. and reference taxa of the genus *Bradyrhizobium*. Posterior probabilities ≥ 0.90 are shown. Bar, expected substitutions per site.

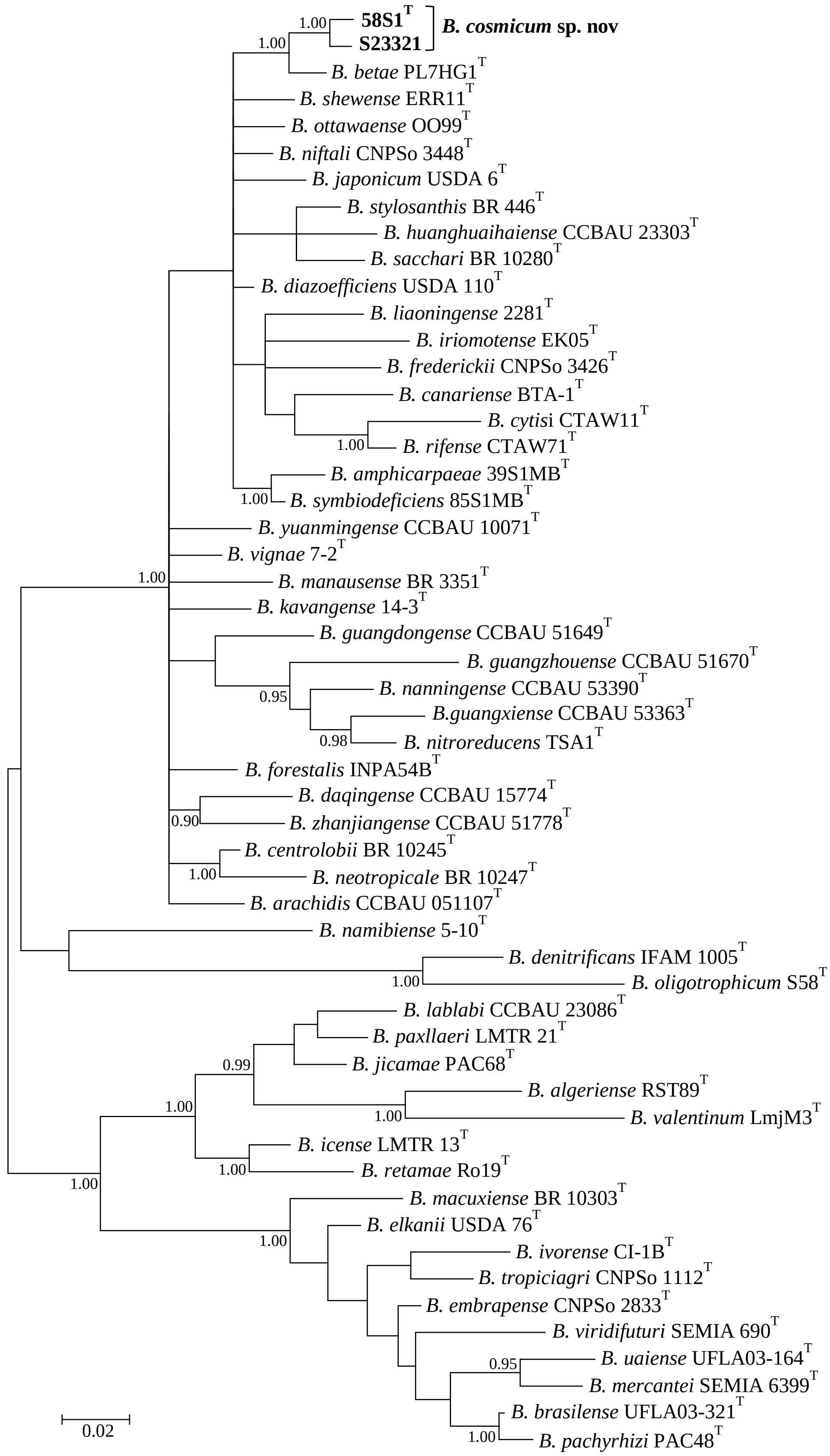

Fig. S3. Bayesian phylogenetic tree (GTR + G + I substitution model) of *gln*II housekeeping gene sequences (519 bp) for *Bradyrhizobium cosmicum* sp. nov. and reference taxa of the genus *Bradyrhizobium*. Posterior probabilities ≥ 0.90 are shown. Bar, expected substitutions per site.

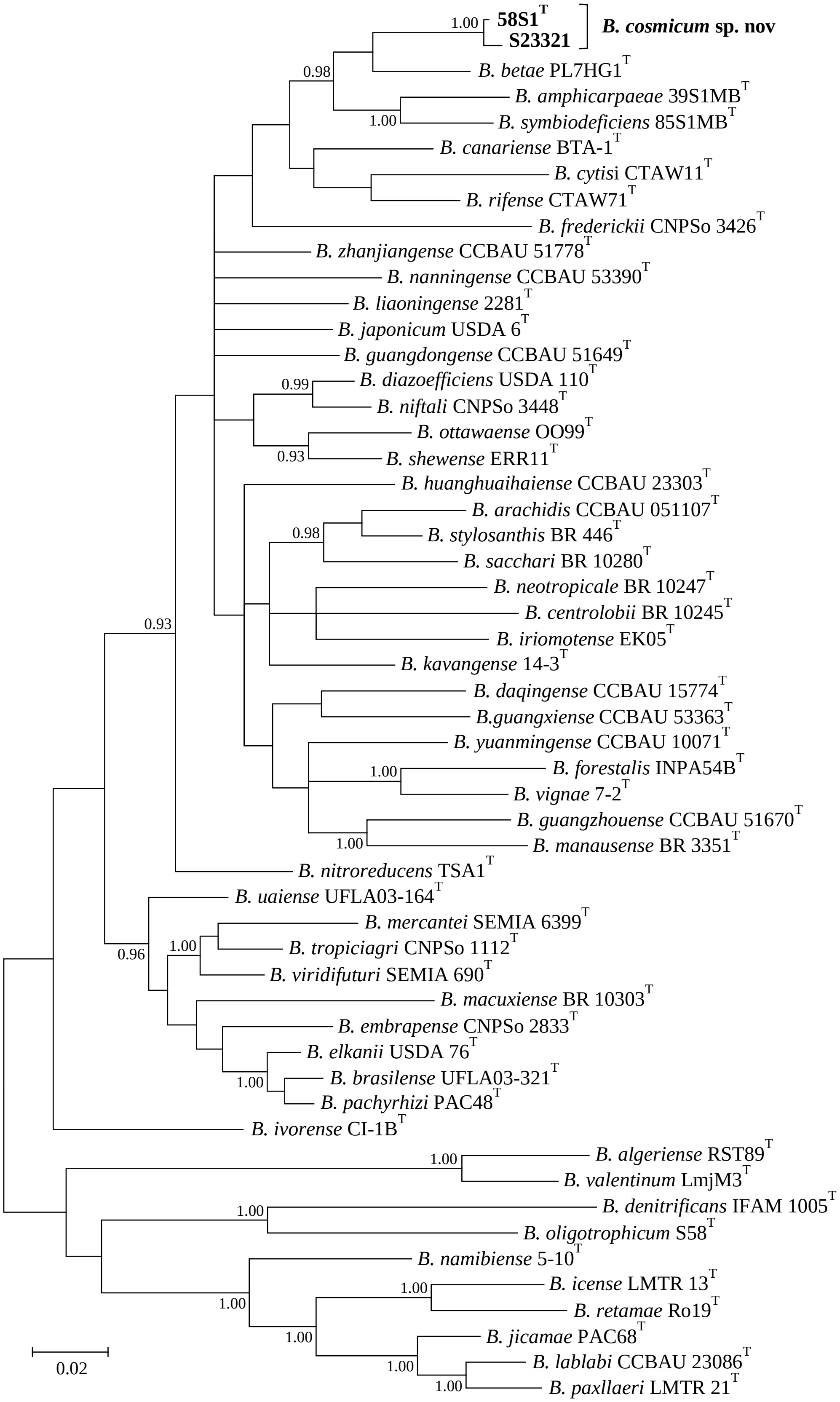

Fig. S4. Bayesian phylogenetic tree (GTR + G + I substitution model) of *gyr*B housekeeping gene sequences (600 bp) for *Bradyrhizobium cosmicum* sp. nov. and reference taxa of the genus *Bradyrhizobium*. Posterior probabilities ≥ 0.90 are shown. Bar, expected substitutions per site.

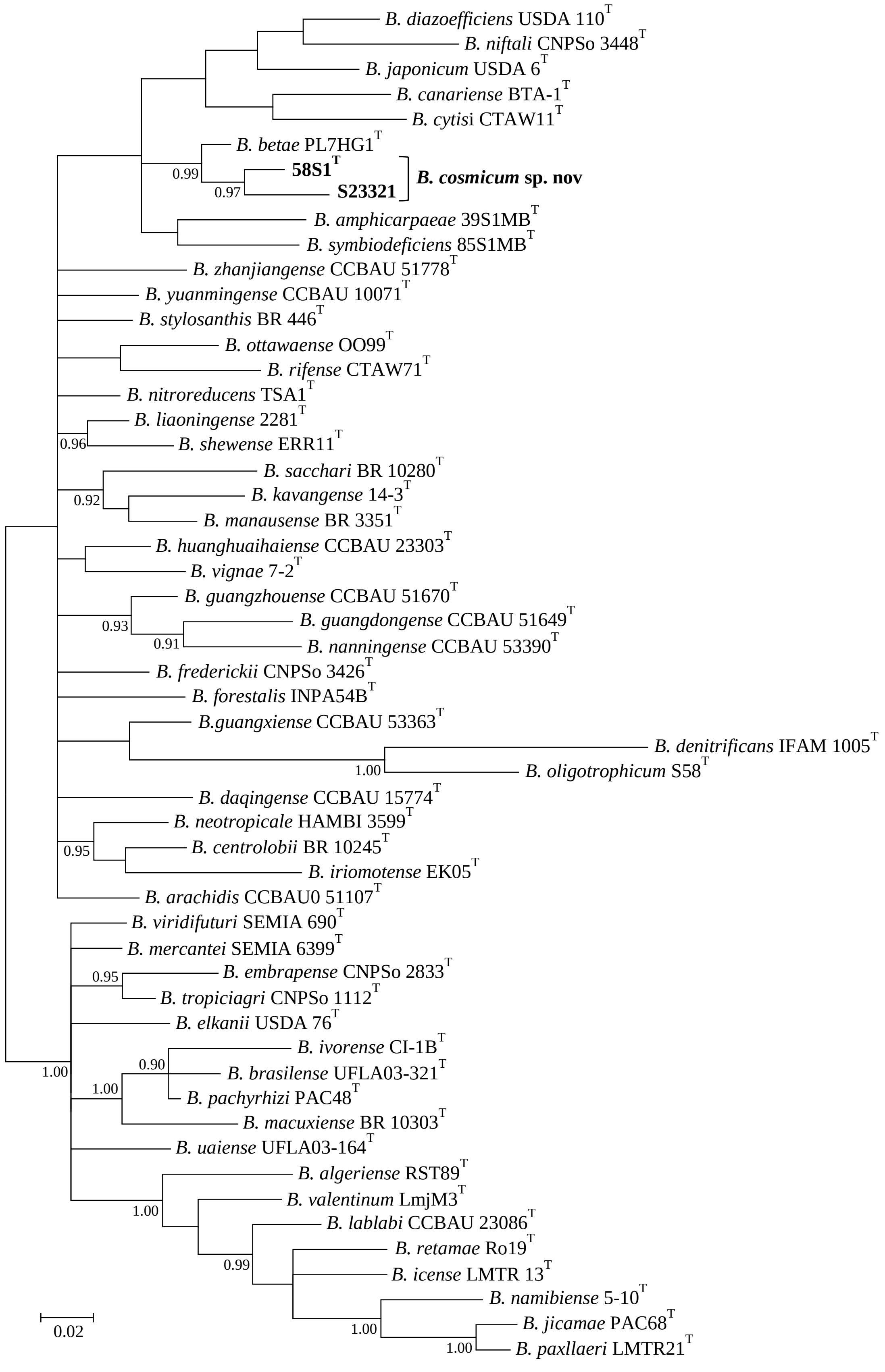

Fig. S5. Bayesian phylogenetic tree (GTR + G + I substitution model) of *rec*A housekeeping gene sequences (417 bp) for *Bradyrhizobium cosmicum* sp. nov. and reference taxa of the genus *Bradyrhizobium*. Posterior probabilities ≥ 0.90 are shown. Bar, expected substitutions per site.

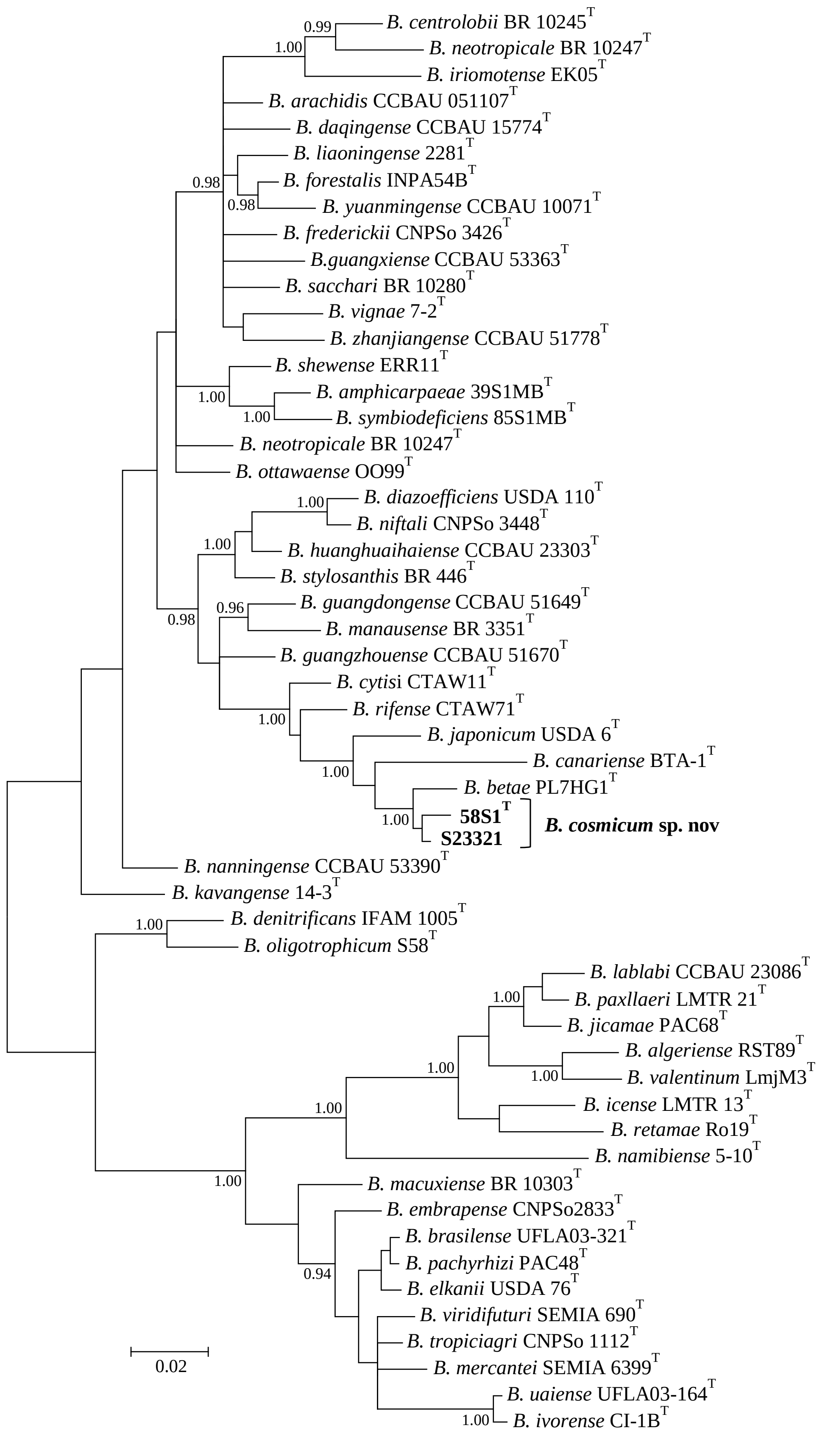

Fig. S6. Bayesian phylogenetic tree (GTR + G + I substitution model) of *rpo*B housekeeping gene sequences (714 bp) for *Bradyrhizobium cosmicum* sp. nov. and reference taxa of the genus *Bradyrhizobium*. Posterior probabilities ≥ 0.90 are shown. Bar, expected substitutions per site.

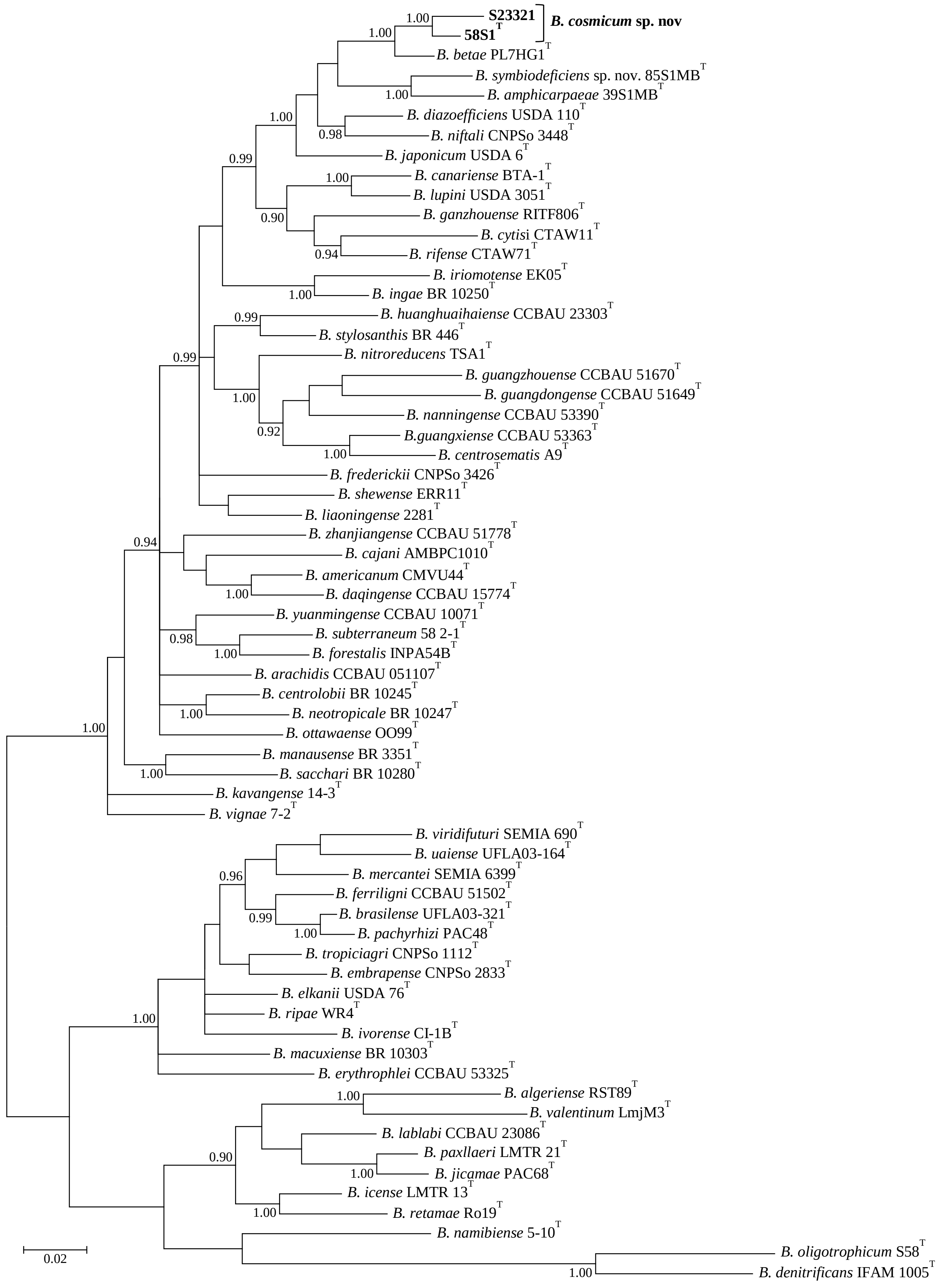

S23321
58S1T
B. cosmicum sp. nov
B. betae PL7HG1T
B. symbiodeficiens sp. nov. 85S1MBT
B. amphicarpaeae 39S1MBT
B. diazoefficiens USDA 110T
B. niftali CNPSo 3448T
B. japonicum USDA 6T
B. canariense BTA-1T
B. lupini USDA 3051T
B. ganzhouense RITF806T
B. cytisi CTAW11T
B. rifense CTAW71T
B. iriomotense EK05T
B. ingae BR 10250T
B. huanghuaihaiense CCBAU 23303T
B. stylosanthis BR 446T
B. nitroreducens TSA1T
B. guangzhouense CCBAU 51670T
B. guangdongense CCBAU 51649T
B. nanningense CCBAU 53390T
B.guangxiense CCBAU 53363T
B. centrosematis A9T
B. frederickii CNPSo 3426T
B. shewense ERR11T
B. liaoningense 2281T
B. zhanjiangense CCBAU 51778T
B. cajani AMBPC1010T
B. americanum CMVU44T
B. daqingense CCBAU 15774T
B. yuanmingense CCBAU 10071T
B. subterraneum 58 2-1T
B. forestalis INPA54BT
B. arachidis CCBAU 051107T
B. centrolobii BR 10245T
B. neotropicale BR 10247T
B. ottawaense OO99T
B. manausense BR 3351T
B. sacchari BR 10280T
B. kavangense 14-3T
B. vignae 7-2T
B. viridifuturi SEMIA 690T
B. uaiense UFLA03-164T
B. mercantei SEMIA 6399T
B. ferriligni CCBAU 51502T
B. brasilense UFLA03-321T
B. pachyrhizi PAC48T
B. tropiciagri CNPSo 1112T
B. embrapense CNPSo 2833T
B. elkanii USDA 76T
B. ripae WR4T
B. ivorense CI-1BT
B. macuxiense BR 10303T
B. erythrophlei CCBAU 53325T
B. algeriense RST89T
B. valentinum LmjM3T
B. lablabi CCBAU 23086T
B. paxllaeri LMTR 21T
B. jicamae PAC68T
B. icense LMTR 13T
B. retamae Ro19T
B. namibiense 5-10T
B. oligotrophicum S58T
B. denitrificans IFAM 1005T
0.02

Supplementary Fig. S7. Bayesian phylogenetic tree (GTR + G + I substitution model) of *rec*A-*gln*II concatenated gene sequences (930 bp) for *Bradyrhizobium cosmicum* sp. nov. and reference taxa of the genus *Bradyrhizobium*. Only posterior probabilities ≥ 0.90 are shown. Bar, expected substitutions per site.

To include all named species of *Bradyrhizobium* in the analysis, it was necessary to trim individual sequence alignment lengths to 411 and 519 bp for *rec*A and *gln*II, respectively.

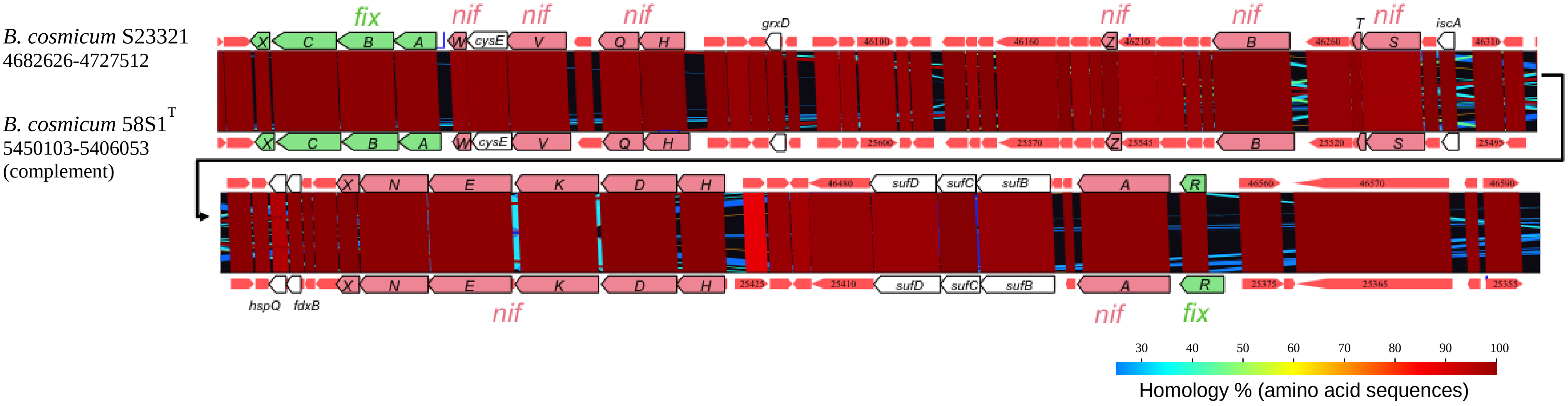

Supplementary Fig. S8: Comparative arrangement of the nitrogen fixation gene cluster of *Bradyrhizobium cosmicum* S23321 and 58S1^{T}. Color coding represents % homology based on amino acid sequences calculated by GenomeMatcher (Ohtsubo Y, Ikeda-Ohtsubo W, Nagata Y, Tsuda M. *BMC Bioinf* 2008; 9:376).

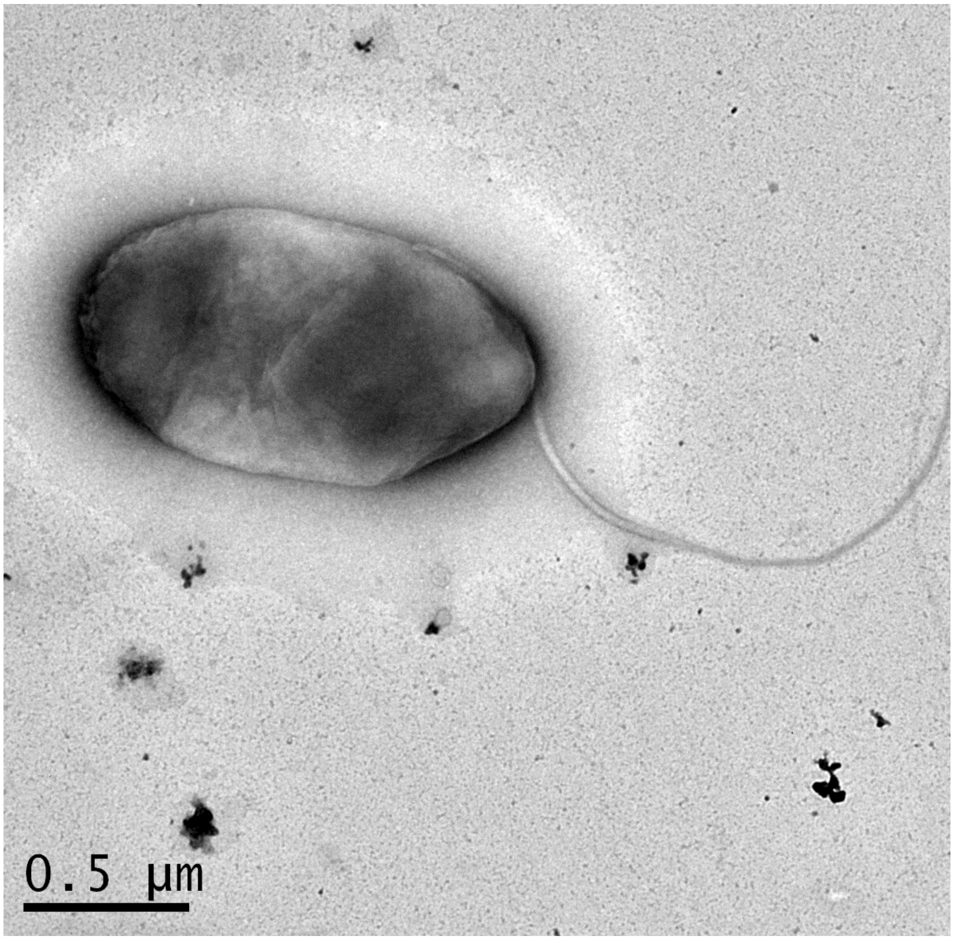

Supplementary Fig. S9. Transmission electron micrograph showing a cell of *B. cosmicum* sp. nov., 58S1^{T} with sub-polar flagellum.
Average cell size, 0.86 x 1.64 µm (based on 10 measurements).
